# Leveraging Implementation Science at the Early-Stage Development of a Novel Telehealth-Delivered Fear of Exercise Program to Understand Intervention Feasibility and Implementation Potential: Feasibility Behavioral Intervention Study

**DOI:** 10.2196/55137

**Published:** 2024-11-12

**Authors:** Andrea T Duran, Robin M Cumella, Miguel Mendieta, Adrianna Keener-Denoia, David López Veneros, Samantha G Farris, Nathalie Moise, Ian M Kronish

**Affiliations:** 1 Center for Behavioral Cardiovascular Health Columbia University Irving Medical Center New York, NY United States; 2 Columbia University School of Nursing New York, NY United States; 3 Department of Psychology Rutgers, The State University of New Jersey Piscataway, NJ United States

**Keywords:** behavioral intervention development, implementation science, acute coronary syndrome, exercise sensitivity, interoceptive exposure, digital health, mobile phone

## Abstract

**Background:**

To increase real-world adoption of effective telehealth-delivered behavioral health interventions among midlife and older adults with cardiovascular disease, incorporating implementation science (IS) methods at earlier stages of intervention development may be needed.

**Objective:**

This study aims to describe how IS can be incorporated into the design and interpretation of a study assessing the feasibility *and* implementation potential of a technology-delivered behavioral health intervention.

**Methods:**

We assessed the feasibility and implementation potential of a 2-session, remotely delivered, home-based behavioral intervention composed of psychoeducation, interoceptive exposure through low-to-moderate intensity walking, interoceptive counseling, and homework (Reducing Exercise Sensitivity with Exposure Training; RESET) among patients with recent acute coronary syndrome (ACS) and some fear of exercise. To assess intervention feasibility, we measured patient protocol adherence, intervention delivery fidelity, and completion of intervention outcome assessments using direct observations, fidelity checklists, surveys, and device-measured physical activity. To assess implementation potential, we measured implementation outcomes (feasibility, acceptability, and appropriateness) using 4-item measures, each rated from the patient perspective on a 1 to 5 Likert scale (1=completely disagree and 5=completely agree; criteria: ≥4=agree or completely agree), and patient-perceived implementation determinants and design feedback using survey and interview data. Interview data underwent thematic analysis to identify implementation determinant themes, which were then categorized into Consolidated Framework for Implementation Research (CFIR) domains and constructs.

**Results:**

Of 31 patients approached during recruitment, 3 (10%) were eligible, enrolled, and completed the study (mean age 46.3, SD 14.0 y; 2/3, 67% male; 1/3, 33% Black; and 1/3, 33% Asian). The intervention was delivered with fidelity for all participants, and all participants completed the entire intervention protocol and outcome assessments. On average, participants agreed that the RESET intervention was feasible and acceptable, while appropriateness ratings did not meet implementation criteria (feasibility: mean 4.2, SD 0.4; acceptability: mean 4.3, SD 0.7; and appropriateness: mean 3.7, SD 0.4). Key patient-perceived implementation determinants were related to constructs in the innovation (design, adaptability, and complexity), inner setting (available resources [physical space, funding, materials, and equipment] and access to knowledge and information), and innovation recipient characteristics (motivation, capability, opportunity, and need) domains of the CFIR, with key barriers related to innovation design. Design feedback indicated that the areas requiring the most revisions were the interoceptive exposure design and the virtual delivery modality, and reasons why included low dose and poor usability.

**Conclusions:**

The RESET intervention was feasible but not implementable in a small sample of patients with ACS. Our theory-informed, mixed methods approach aided our understanding of what, how, and why RESET was not perceived as implementable; this information will guide intervention refinement. This study demonstrated how integrating IS methods early in intervention development can guide decisions regarding readiness to advance interventions along the translational research pipeline.

## Introduction

### Background

Cardiovascular disease (CVD) is the leading cause of mortality in the United States, accounting for >860,000 deaths in 2019 and an estimated cost of >US $200 billion annually in health care services, medications, and lost productivity [1,2]. As the population of older adults (aged ≥65 years) continues to grow, the prevalence of CVD is expected to increase as adults transition from midlife to older adulthood [2,3]. To combat the public health and economic burden of CVD, strong emphasis has been placed on developing interventions that target modifiable health behaviors that contribute to cardiovascular health, such as physical activity, as well as underlying behavioral mechanisms (eg, self-efficacy and fear) [4-9]. In recent years, and particularly since the COVID-19 pandemic, digital health technologies (eg, telehealth, telemedicine, mobile health, and remote patient monitoring) have emerged as a cornerstone to the delivery, monitoring, and measurement of behavioral interventions to improve CVD [10-13]. Although encouraging, the appropriate development, evaluation, and implementation of digital health interventions to improve heart-healthy lifestyle behaviors in midlife to older adult populations as well as their integration into real-world settings require further investigation [14,15].

To facilitate successful behavioral health-related intervention development, translational research frameworks, such as the National Institutes of Health (NIH) Stage Model [16], NIH’s Obesity-Related Behavioral Intervention Trials Model [17,18], and Multiphase Optimization Strategy [19], have been generated to guide the development of behavioral interventions toward large-scale dissemination and implementation [20]. To supplement the translational research pipeline, experts in behavioral intervention development have posited that principles and methods from the field of implementation science (IS)—a field that uses theory, models, and frameworks to understand the factors that influence and strategies that enable a timely and successful uptake of a clinical innovation across diverse real-world settings [21,22]—be integrated at earlier stages of intervention development (ie, before implementation) [23]. To date, efforts to blend elements of behavioral intervention development and IS have emerged in the form of hybrid effectiveness-implementation studies, a framework that bridges the gap between clinical effectiveness and implementation and has been widely adopted within the field of IS [24,25]. However, there are few, if any, applied research examples on how to incorporate IS methods and principles at earlier stages of behavioral intervention development (eg, intervention generation, refinement, feasibility, and pilot testing), an approach that may enhance the potency and implementation potential of digital health behavioral interventions among midlife and older adults with CVD in real-world settings. Here, we describe how we blended elements of a feasibility study and implementation research to conduct, what we propose as, a hybrid “feasibility-implementation” study at the early stages of behavioral intervention development.

### Objectives

This study aims to describe how IS methods and principles were incorporated into the design of an early-stage feasibility study testing the feasibility *and* implementation potential of a technology-delivered behavioral intervention. To our knowledge, this is one of the first studies to apply a theory-informed, mixed methods approach at the early stages of behavioral intervention development in patients with acute coronary syndrome (ACS), a vulnerable, predominantly midlife and older adult CVD population.

## Methods

### Conceptual Model

**Figure 1:**
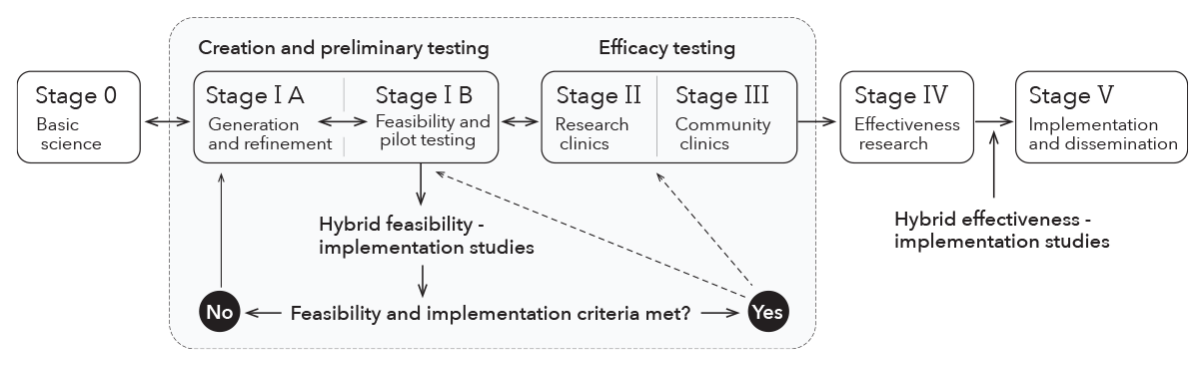
Conceptual model for integrating implementation science methods into the earlier stages of behavioral intervention development.

This work was guided by the NIH Stage Model for behavioral intervention development, an iterative, recursive, and multidirectional model created to facilitate the scientific development of behavioral interventions [16]. The NIH Stage Model is intended to help intervention developers and practitioners identify where a behavioral intervention lies within the intervention development process as well as inform best next steps to ensure that the intervention reaches its maximum effectiveness and implementation potential in real-world settings. Accordingly, the NIH Stage Model includes the following 6 stages of behavioral intervention development: basic science (stage 0); intervention generation, refinement, modification, and adaptation (stage IA); feasibility and pilot testing (stage IB); traditional efficacy testing (stage II); efficacy testing with real-world providers (stage III); effectiveness research (stage IV); and dissemination and implementation research (stage V). As widely documented, persistent evidence-to-practice gaps exist as interventions transition from stage IV to stage V, a gap that has been the primary focus of IS as a field over the past 2 decades [22]. However, as posited by NIH Stage Model pioneers, there is also a need to address implementation issues as early as possible in the intervention development process and before the intervention is studied in trials at later stages (ie, before effectiveness evaluation) [23]. Therefore, we propose that the methods (eg, theory, models, and frameworks), outcomes (eg, acceptability, adoption, appropriateness, costs, feasibility, fidelity, penetration, and sustainability), and principles (eg, understanding behavioral and contextual implementation determinants) underpinning the field of IS to also be considered at stages I, II, and III of the NIH Stage Model (Figure 1). Our proposed formulation is consistent with the NIH Stage Model’s iterative and multidimensional (vs linear, sequential ordering) nature [16]. Moreover, it aligns with the notion that implementability is an important consideration even during the early stages of intervention development [23,26,27].

Modeling our approach after the hybrid effectiveness-implementation studies proposed by Curran et al [24,25], which combine research questions concerning specific aspects of intervention effectiveness and implementation strategies within the same study, we propose a hybrid “feasibility-implementation” study, which combine research questions concerning intervention feasibility (eg, patient and therapist adherence to intervention protocol) and intervention implementation potential (eg, contextual barriers and key stakeholder perceptions of feasibility, acceptability, and appropriateness) within the same study (Figure 1). Of note, a feasibility study refers to whether an intervention or trial can be successfully conducted, whereas a pilot study is a type of feasibility study with the special design feature of being a smaller version of a planned or proposed behavioral trial (ie, randomized) [28,29]. Information gleaned from our proposed “feasibility-implementation” model can help intervention developers navigate earlier stages of the intervention development process (ie, stage IB pilot study; stage II or III efficacy study) based on predefined criteria, while simultaneously providing insights as to what, how, and why implementation succeeded or failed and theory-informed, mixed methods data needed to generate new materials or refine an intervention, an approach that has the potential to boost the effects and implementation of behavioral interventions at later stages of behavioral intervention development (ie, stage IV effectiveness and stage V implementation and dissemination). Furthermore, as intervention development is neither prescriptive nor linear [16], this model can be applied to interventions that have proven to be efficacious or effective to further develop or adapt as needed for real-world implementation.

### Study Design

From January to August 2021, we created a remote behavioral intervention designed to reduce exercise sensitivity through exposure training (Reducing Exercise Sensitivity with Exposure Training; RESET) among patients with ACS (stage IA). Here, we provide an applied example of a “feasibility-implementation” study that aimed to simultaneously evaluate intervention feasibility (aim 1) and implementation potential (aim 2) of RESET among patients with ACS at NIH stage IB of behavioral intervention development. Findings from this feasibility study determined whether the RESET intervention should move forward with a stage 1B pilot randomized trial or move back to stage 1A for further intervention refinement or generation of new materials according to predefined criteria. Details of the feasibility study and RESET intervention have been registered on ClinicalTrials.gov (NCT05099926). According to the recent guidelines for reporting behavioral interventions [30], the Template for Intervention Description and Replication checklist and guidelines were followed when describing the RESET intervention [31].

### Study Setting, Patient Population, and Eligibility Criteria

This study was conducted by the Columbia Roybal Center for Fearless Behavioral Change at Columbia University Irving Medical Center (CUIMC) between September 2021 and February 2023. Patients were recruited from inpatient and outpatient services at NewYork-Presbyterian Hospital and CUIMC. A diagnosis of ACS (ie, myocardial infarction or unstable angina) based on the *International Classification of Diseases, Tenth Revision* codes in the patient’s electronic health record within the past 12 months was the primary inclusion criteria [32]. To be included, participants also had to be aged ≥18 years and score >1 (*sometimes*, *often*, or *very often*) on at least 1 item from the 4-item Aversive Cognitions about Physical Activity Scale [33] or score >1 (*some, much, or very much*) on at least 1 item from the 18-item Exercise Sensations Questionnaire (ESQ-18) [34]. The exclusion criteria were as follows: (1) unable to speak and read English, (2) lack access to an electronic tablet or smartphone (iPhone or Android), (3) medical or psychiatric impairment or structural home environment constraints that would prevent safe or adequate participation, (4) unable to comply with the protocol due to cognitive or psychiatric reasons, or (5) unavailable for follow-up.

### Recruitment, Screening, and Enrollment

As the study overlapped with social distancing measures during the COVID-19 pandemic, all study procedures took place remotely. Potential participants were identified through several methods: (1) referral from treating clinicians (eg, physicians, physician assistants, and physical therapists); (2) research studies at our center where participants with ACS International Classification of Diseases, Tenth Revision codes agreed to be contacted about future study opportunities; and (3) electronic health record data retrieval of individuals hospitalized at NewYork-Presbyterian Hospital and CUIMC in the past year, followed by physician approval to contact the patient. Research personnel contacted potentially eligible participants by telephone and obtained verbal consent to complete a battery of questionnaires to confirm eligibility (eg, electronic tablet or smartphone; internet access; adequate home environment; and elevated exercise sensitivity). Once eligibility was confirmed, patients were enrolled in the study.

### Pre- and Postintervention Visits

Following enrollment, participants (1) completed a battery of baseline questionnaires via phone call; (2) received study materials via mail; and (3) completed a preintervention preparation video visit via an encrypted, Health Insurance Portability and Accountability Act–compliant, web-based, videoconferencing application (ie, Zoom [Zoom Video Communications]). Following intervention completion, participants completed (1) a battery of postintervention questionnaires and (2) a semistructured exit interview via phone call.

Materials included a Fitbit InspireHR (Fitbit Inc; to measure heart rate and physical activity), a tripod (to secure and angle the participant’s electronic device), a measuring tape and 2 small agility cones (to set up the walking course), the Borg Category-Ratio 10 scale (to assess perceived exertion during intervention visits), and a physical activity journal (for homework). For the preintervention video visit, research personnel assisted participants with the download of the Fitbit app onto their smartphone, setup and syncing of the Fitbit InspireHR device, setup of the home environment and electronic devices for the intervention walking course (ie, ≥6 feet of flat, unobstructed surface and electronic device placement via tripod to safely monitor participants while they walk), and a brief practice session of the walking activity to familiarize participants with the intervention visits. Details of the materials and instructions needed to set up the walking course and prepare for the walking activity are provided in Multimedia Appendix 1.

### Intervention

#### Overview

**Figure 2:**
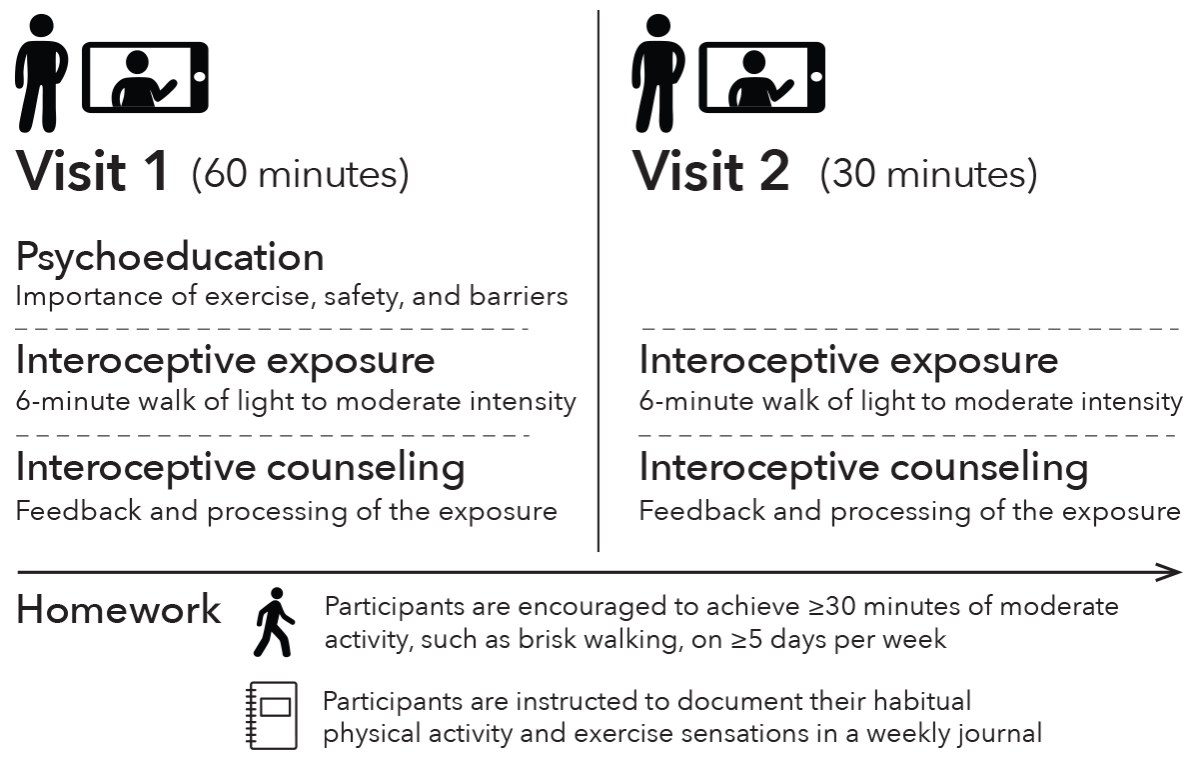
Overview of the Reducing Exercise Sensitivity with Exposure Training intervention visits and components.

RESET is a multicomponent, technology-delivered, at-home, 2 visit intervention that involves psychoeducation; a brief (6 min), low-to-moderate intensity walking session to safely expose participants to averse physical sensations (ie, interoceptive exposure); interoceptive counseling to understand and process physical sensations (eg, rapid heart rate); and homework (Figure 2). RESET is designed to reduce exercise sensitivity (ie, fear of exercise sensations) and, in turn, improve participation in exercise-based secondary prevention guidelines (cardiac rehabilitation and physical activity). Our behavioral intervention targeted exercise sensitivity as our behavioral mechanism of action because survivors of ACS may misattribute physical sensations experienced during exercise (eg, increased heart rate, shortness of breath, and fatigue) as dangerous, intolerable, or similar to sensations experienced or attributed to their ACS event [35]. As a result, patients may avoid situations and activities prescribed as part of their secondary prevention treatment (ie, exercise) that prompt these physical sensations or terminate physical activity at the first sign of discomfort [34-36].

The first intervention visit (video visit 1; approximately 60 min) included psychoeducation, interoceptive exposure, and interoceptive counseling. The second intervention visit (video visit 2; approximately 30 min) only included interoceptive exposure and counseling. For homework, participants were instructed to document their weekly physical activity and accompanied exercise sensations in a journal throughout the duration of the intervention. Each RESET intervention visit occurred once or twice per week based on participant preference. Given the time frame in which the intervention was developed (ie, COVID-19 pandemic) as well as persistent barriers (eg, facility location, transportation, and scheduling) pertinent to behavioral health intervention uptake among patients with cardiac issues [37], the use of digital health technologies to remotely deliver and monitor the intervention was prioritized. Intervention materials and procedures were designed by a clinical exercise physiology and IS expert (ATD), with additional input provided by a clinical psychology expert (SGF), and administered by trained clinical research coordinators and a research nurse (RMC, MM, and DLV). Details of each intervention component are provided in Multimedia Appendix 2 [33,34,38-42], with brief descriptions provided in subsequent sections. Changes made to the protocol throughout the study and the rationale for each change are outlined in Table S1 in Multimedia Appendix 2.

#### Psychoeducation

Psychoeducation content covered topics related to exercise and cardiac rehabilitation (eg, importance, safety, and barriers), exercise sensitivity (eg, avoidance behaviors and biological basis), and interoceptive exposure and counseling (eg, rationale and approach vs avoidance behaviors). These topics were selected to ensure that the participant had the foundational knowledge about the target mechanism (ie, exercise sensitivity) through which the intervention aims to improve the target behavior (eg, exercise) as well as ensure that the participant understands the role of select intervention components (eg, interoceptive exposure and counseling) to address the target mechanism (ie, exercise sensitivity). To facilitate psychoeducation content, participants were guided through a Microsoft PowerPoint presentation with relevant text and visuals (Multimedia Appendix 3).

#### Interoceptive Exposure

A gradual 6-minute walk (G6MW; ie, 6-min bout of light-to-moderate intensity physical activity) served as a low-risk form of remotely monitored interoceptive exposure. To align with the standard of care physical activity guidelines for secondary prevention [38], participants were instructed to walk at a cadence between 50 (light intensity) and 100 steps per minute (moderate intensity) [39]. Stepping cadence was remotely monitored and regulated with a metronome. Before the G6MW, participants were asked to identify and rate how distressing or intense (0-10 scale, where 0=not at all distressing or intense and 10=extremely distressing or intense) anticipated exercise sensations would be during the walking activity. Throughout the G6MW, research personnel measured the participants’ ratings of perceived exertion using the Borg Category-Ratio 10 scale (0=nothing at all and 10=extremely strong [“maximal”]) at 1-minute intervals [42]. Immediately after the G6MW, participants were instructed to sit down in a chair and rest for 3 to 5 minutes. Heart rate was measured before (eg, rest) and after (eg, recovery) the G6MW via Fitbit InspireHR device. Importantly, the goal of the activity was not to deliver exercise, per se, but rather to safely generate exposure to sensations that could be experienced as aversive (eg, shortness of breath and rapid heart rate) in our patient population [41].

#### Interoceptive Counseling

Participants identified and rated how distressing or intense (0-10 scale, where 0=not at all distressing or intense and 10=extremely distressing or intense) exercise sensations were during the interoceptive exposure activity (ie, G6MW). Next, a member of the research team reviewed and compared pre- and postinteroceptive exposure ratings with participants (Figure S1 in Multimedia Appendix 2) to help facilitate the feedback and processing of the exposure (eg, *surprising*: “It was not as bad as I expected”); *different*: “I thought I would feel X, but I actually felt Y”; and *similar*: “It was just as I expected...uncomfortable”).

#### Homework

Participants were encouraged (but not required) to achieve ≥30 minutes of moderate aerobic activity, such as brisk walking, on ≥5 days per week. To help participants self-identify and reflect on sensations that are normal (vs distressing) in response to physical activity in the real world (vs monitored intervention sessions), the participants were instructed to document their habitual physical activity (ie, date, time of day, duration, and modality), exercise sensations, and perceptions of exercise sensation distress in a weekly journal starting after video visit 1 and ending the week of video visit 2, as well as sync their Fitbit InspireHR device with the Fitbit app daily. A member of the research team spent approximately 5 minutes at the end of video visit 1 to familiarize participants with the weekly journal instructions and content (Figure S2 in Multimedia Appendix 2) as well as provided examples to ensure participant comprehension. Journal entries were reviewed with research personnel at the final intervention visit (video visit 2). Fitbit data were confirmed by research personnel upon study completion.

### Measures

#### Overview

Table 1 provides an overview of the primary, secondary, and exploratory measures obtained to assess intervention feasibility (aim 1) and implementation potential (aim 2); a brief description of each measure; and the time points at which each measure was captured. The conceptual framework for implementation outcomes proposed by Proctor et al [43] (ie, indicators of implementation success that represent necessary preconditions for attaining desired changes in behavior and other clinical outcomes) [43] was used to define our implementation indices and success.

**Table 1:** Details regarding study measures, measure type, description, and assessment time frame for each study aim.

Study aim	Measure type	Measure	Brief description	Assessment time frame
Aim 1	Primary	Intervention adherence	Proportion of participants who completed their intervention visits as intended	From baseline to study completion
Aim 1	Primary	Intervention fidelity	Proportion of participants who received their intervention visits administered as intended	From baseline to study completion
Aim 1	Primary	Intervention outcome assessment	Proportion of participants who completed their outcome assessments upon intervention completion	Study completion
Aim 2	Primary	Intervention feasibility	Patient-perceived feasibility of the intervention (4-item Feasibility of Intervention Measure)	Study completion
Aim 2	Primary	Intervention acceptability	Patient-perceived acceptability of the intervention (4-item Acceptability of Intervention Measure)	Study completion
Aim 2	Primary	Intervention appropriateness	Patient-perceived appropriateness of the intervention (4-item Intervention Appropriateness Measure)	Study completion
Aim 2	Secondary	Implementation determinants	Patient-perceived barriers and facilitators to intervention implementation (audio-recorded interview)	Study completion
Aim 2	Secondary	Intervention design	Participant feedback on (audio-recorded interview) and satisfaction with (5-point Likert scale) the RESET^a^ intervention design	Study completion
Aim 1	Exploratory	Change in exercise sensitivity	Pre- to postintervention change in exercise sensitivity (18-item Exercise Sensations Questionnaire)	Baseline and study completion
Aim 1	Exploratory	Change in physical activity levels	Pre- to postintervention change in physical activity levels (7-item International Physical Activity Questionnaire-Short Form)	Baseline and study completion

^a^RESET: Reducing Exercise Sensitivity with Exposure Training.

#### Primary Feasibility and Implementation Outcome Measures

##### Intervention Adherence (Aim 1)

Adherence to the intervention was calculated as the proportion of intervention participants who attended and completed all RESET intervention visits [44,45]. An intervention visit was defined as complete when a participant engaged in ≥90% of each intervention component (ie, psychoeducation [eg, audiovisual communication established throughout education and responded to prompts], interoceptive exposure [eg, wore Fitbit and footwear; walking course appropriately setup in home environment; completed G6MW; and completed pre-, during, and post-G6MW assessments—anticipated or experienced sensations, ratings of perceived exertion, distress, and heart rate], interoceptive counseling [eg, audiovisual communication established throughout counseling, responded to prompts about walking sensations, ratings, and avoidance behaviors], and homework [eg, completed ≥1 physical activity and exercise sensations journal entry]) as intended by the intervention protocol [44].

##### Intervention Delivery Fidelity (Aim 1)

Fidelity of intervention delivery was calculated as the proportion of participants who had their intervention visit components administered as intended by the interventionist per protocol procedures [44,46-48]. An intervention component was defined as being administered as intended when ≥90% of protocol items were marked as completed via an intervention fidelity checklist. The intervention fidelity checklist was completed by research personnel during each intervention session via direct observation of the interventionist [44,49].

##### Completion of Intervention Outcome Assessments (Aim 1)

Feasibility of completing intervention outcome assessments was calculated as the proportion of intervention participants who completed their outcome assessments upon intervention completion [44,45,47]. Outcome assessments were defined as complete when ≥90% of data (eg, nonmissing questionnaire items, interview responses, or Fitbit data) were collected for the following outcome measures: exercise sensitivity, self-reported physical activity, device-measured physical activity, implementation outcomes (feasibility, acceptability, and appropriateness), and implementation determinants. These outcome measures were prioritized, as they would serve as the primary and secondary outcome measures tested in future trials.

##### Intervention Feasibility (Aim 2)

Participants’ perception that the intervention was feasible (ie, intervention can be used or carried out within a given agency or setting) was assessed using the valid, reliable, and pragmatic 4-item Feasibility of Intervention Measure (FIM) [40]. Each item was rated on a 1 to 5 Likert scale (1=completely disagree and 5=completely agree), and the mean of all item responses (score range 1-5) was used, with higher scores indicating greater feasibility.

##### Intervention Acceptability (Aim 2)

Participants’ perception that the intervention was acceptable (ie, agreeable, palatable, or satisfactory) was assessed using the valid, reliable, and pragmatic 4-item Acceptability of Intervention Measure (AIM) [40]. Each item was rated on a 1 to 5 Likert scale (1=completely disagree and 5=completely agree), and the mean of all item responses (score range 1-5) was used, with higher scores indicating greater acceptability.

##### Intervention Appropriateness (Aim 2)

Participants’ perception that the intervention was appropriate (ie, perceived fit, relevance, or compatibility of the intervention for a given patient or a particular issue) was assessed using the valid, reliable, and pragmatic 4-item Intervention Appropriateness Measure (IAM) [40]. Each item was rated on a 1 to 5 Likert scale (1=completely disagree and 5=completely agree), and the mean of all item responses (score range 1-5) was used, with higher scores indicating greater appropriateness.

#### Feasibility and Implementation Criteria

Feasibility was achieved if each of the following criteria were met: (1) ≥90% of participants completed all RESET intervention visits (adherence), (2) ≥90% of participants received intervention components as intended (fidelity), and (3) ≥90% of participants completed their outcome assessments upon intervention completion (outcome assessment) [44]. Implementation potential was achieved if each of the following criteria were met: (1) participants reported, on average, a mean FIM score of ≥4 (agree or completely agree; feasibility); (2) participants reported, on average, a mean AIM score of ≥4 (agree or completely agree; acceptability); and (3) participants reported, on average, a mean IAM score of ≥4 (agree or completely agree; appropriateness) [40,50].

#### Secondary Implementation Measures

##### Implementation Determinants and Design Feedback (Aim 2)

Participant-perceived barriers and facilitators to using the intervention and intervention design feedback were assessed using a semistructured audio-recorded exit interview with open-ended questions [51,52]. The interview guide is included in Multimedia Appendix 2. To elicit implementation determinants, participants were asked about the challenges and concerns they experienced while participating in RESET as well as what would have helped them participate to the fullest extent. To elicit design feedback, participants were asked about what they liked the most and least about RESET and what they would change.

##### Satisfaction With Each Intervention Component (Aim 2)

Participant-perceived satisfaction with the intervention was assessed using a 1-item question per intervention component (ie, psychoeducation, interoceptive exposure, interoceptive counseling, and homework) [53]. Each item was rated on a 1 to 5 Likert scale (1=very dissatisfied and 5=very satisfied), with higher scores indicating greater satisfaction.

#### Exploratory Feasibility Measures

##### Pre- to Postintervention Change in Exercise Sensitivity (Aim 1)

Exercise sensitivity was measured using the 18-item ESQ-18 [35]. Each ESQ-18 item reflects fear and anxiety of various bodily sensations and was rated on a 0 to 4 Likert scale (0=not at all and 4=very much) based on agreement with each statement. Scores of all the items were summed (score range 0-72), with higher scores indicating greater fear of exercise. Pre-to-post program changes in exercise sensitivity were calculated by subtracting the preintervention ESQ-18 score from the postintervention ESQ-18 score.

##### Pre- to Postintervention Change in Physical Activity Levels (Aim 1)

Self-reported physical activity was measured using the 7-item International Physical Activity Questionnaire-Short Form (IPAQ-SF). IPAQ-SF items elicit participants’ last 7-day recall of the days and time (minutes) spent in physical activities of different intensities (vigorous, moderate, and walking) as part of their everyday lives. Each item response was inserted into an IPAQ-SF scoring tool [54] to estimate the total volume of weekly physical activity in metabolic equivalent task (MET) minutes per week.

### Data Analysis

#### Quantitative and Qualitative Data Analysis

Given that this NIH stage 1B feasibility study [16] was, by design, not powered to test the effect of the RESET intervention on outcomes, analyses were conducted on the data collected for the purposes of demonstrating (1) intervention feasibility and (2) intervention implementation potential. Descriptive statistics, including frequencies and means (SDs), were used to describe quantitative study outcomes. To analyze qualitative data, audio-recorded exit interviews were transcribed verbatim and entered into NVivo (version 14; QSR International) for thematic analysis organization. Transcripts were coded for barriers, facilitators, and design feedback by 2 study team members (AK-D and ATD) using a combination of inductive and deductive analysis via a 6-phase process and qualitative Consolidated Criteria for Reporting Qualitative Studies (COREQ) research checklist [55,56]. Each determinant theme (ie, barriers and facilitators) was then categorized into relevant theoretical domains and constructs of the Consolidated Framework for Implementation Research (CFIR) 2.0 (ie, 39 constructs within 5 domains [eg, inner setting, outer setting, individual characteristics, innovation, and implementation process]) [57-60]. Each design feedback theme was then categorized into relevant intervention components (ie, psychoeducation, interoceptive exposure, interoceptive counseling, and homework) and delivery modality (ie, video visits) and mapped onto patient-perceived satisfaction ratings to inform future intervention refinement or generation. Transcripts were also reviewed and coded for themes related to feasibility, appropriateness, and acceptability to further understand implementation outcome survey responses (ie, FIM, AIM, and IAM).

#### Intervention Feasibility and Implementation Analysis

Evaluation of feasibility and implementation end points and criteria informed the decision on whether to move forward with a stage IB pilot study with a randomized trial design (primary trial feasibility outcomes: recruitment, fidelity, and outcome assessment; primary implementation potential outcomes: participant-perceived acceptability and appropriateness) or move back to stage 1A for further intervention refinement or generation of new materials. If all feasibility and implementation criteria were met, then the RESET intervention was deemed feasible and implementable among survivors of ACS, and the decision to move forward with a stage IB pilot study with a randomized trial design was supported. If all criteria for 1 outcome (ie, feasibility) were met, but not the other (ie, implementation), then the decision to move back to stage 1A for further intervention refinement or generation of new materials was supported. In each decision, information gleaned from implementation determinants and design feedback was used to either refine the intervention or develop appropriate implementation strategies to be tested at future stages of intervention development (ie, stage II or III).

### Ethical Considerations

Ethics approval was obtained from the CUIMC institutional review board (IRB-AAAT6275), and all participants provided verbal informed consent. Participants who completed all components of the study received a total compensation of US $150 and were permitted to keep the study-provided Fitbit InspireHR. Compensation was uploaded to a Bank of America Paycard in two installments: (1) US $50 after completing the preintervention preparation video visit and (2) US $100 following successful completion of the intervention visits, postintervention questionnaires, and audio-recorded exit interview. Data were anonymized, and all personal identifiers were removed.

## Results

### Study Enrollment and Participant Characteristics

The CONSORT (Consolidated Standards of Reporting Trials) guidelines for randomized pilot and feasibility trials [61] were referenced to inform the reporting of our nonrandomized feasibility study results. Figure S1 in Multimedia Appendix 4 presents the study CONSORT flow diagram. From September 2021 to December 2022, we contacted 31 patients with ACS, of which 8 (26%; mean 53.9, SD 12.0 y; 6/8, 75% male; 3/8, 38% Black; and 1/8, 13% Hispanic) provided verbal consent to participate. Of those, 4 (50%; mean 48.8, SD 12.4 years; 3/4, 75% male; 2/4, 50% Black; 1/4, 25% Asian; 4/4, 100% >high school education; and 2/4, 50% living alone) were eligible and enrolled. Among the 23 participants who were approached but did not provide verbal consent, 13 (57%) declined to participate because they reported being too busy (8/13, 61%), not being interested (4/13, 31%), or wanting physician endorsement (1/13, 8%). Participants who enrolled in the study were predominantly men, middle aged, educated, and racially diverse. Among those enrolled, 75% (3/4) of the participants (mean 46.3, SD 14.0 y) initiated the intervention (ie, intervention participants), and the remaining participant (1/4, 25%) was lost to follow-up after baseline assessment and before receiving any intervention components. All 3 intervention participants had access to a tablet and smartphone, and all 3 participants preferred to use their smartphone as their personal electronic device for the intervention.

### Primary Feasibility and Implementation Outcome Results

All intervention feasibility, but not implementation, criteria were met (Table 2). All 3 intervention participants completed 100% of both RESET intervention visits, and 100% of each intervention visit was administered as intended by the interventionist, according to the research protocol. Similarly, all 3 intervention participants completed their pre- and postintervention assessments (exercise sensitivity and physical activity), daily device-measured physical activity assessments with data successfully uploaded to their Fitbit app, postintervention questionnaires (feasibility, acceptability, appropriateness, and satisfaction), and exit interview (barriers, facilitators, and design feedback). On average, participants agreed that the RESET intervention was feasible and acceptable, while participants neither agreed nor disagreed that the intervention was appropriate. Relevant quotes for feasibility, appropriateness, and acceptability are presented in Table S1 in Multimedia Appendix 4. Key reasons why the intervention lacked high participant-perceived appropriateness were related to the perceived fit of the interoceptive exposure design relative to patient needs (“The walking part, I think was a little slow for me. It was a little slow. I could have walked a little bit faster.” [ID_449]) and target population (“...I think [the intervention] would be more appropriate for someone who is somewhat reluctant.” [ID_439]).

**Table 2:** Primary feasibility and implementation outcomes for intervention participants (n=3).

Outcome type and measure		Values	Criteria met
**Feasibility (participants)** **, n (%)**			
	Intervention adherence	3 (100)	Yes
	Intervention fidelity	3 (100)	Yes
	Intervention outcome assessment	3 (100)	Yes
**Implementation (score), mean (SD)**			
	Intervention feasibility	4.2 (0.4)	Yes
	Intervention acceptability	4.3 (0.7)	Yes
	Intervention appropriateness	3.7 (0.4)	No

### Secondary Intervention Implementation Results

#### Implementation Determinants

The determinants of RESET implementation categorized by the CFIR domains and constructs are presented in Table S1 in Multimedia Appendix 5. The determinant themes to RESET implementation were mapped onto the innovation, individual role (ie, innovation recipient) and characteristics, and inner setting domains. Key barriers in the innovation domain (construct) included low interoceptive exposure dose (design), inability to tailor the intervention to participant needs and preferences (adaptability), and difficulty of virtual delivery modality (complexity), while key facilitators (construct) included clarity of intervention instructions (design), positive perception of intervention design and equipment (design), and ease of using intervention materials and equipment (complexity). Key facilitators in the innovation recipient characteristics domain (construct) included the potential of the intervention to increase self-efficacy (capability), provide external motivation (motivation), and offer convenience via the virtual delivery modality (opportunity), while a key barrier (construct) was an inappropriate patient population (need). Key facilitators in the inner setting domain (construct) included access to intervention materials (available resources [funding, materials, and equipment]) and high quality of intervention support (access to knowledge and information), while a key barrier (construct) was restricted home environment capacity (available resources [space]).

#### Intervention Design Feedback

Key design suggestions as well as satisfaction ratings for each intervention component are presented in Table S2 in Multimedia Appendix 4. When asked about RESET intervention design, all participants reported interoceptive exposure design (ie, light dose, short walking course length, and inability to tailor to participant needs or preferences) as what they liked the least, while there was variability in what participants reported liking the most (ie, convenience, focus on physical activity and fear of exercise, and Fitbit). Feedback on changes to the intervention design was predominantly related to improvements in virtual delivery modality and interoceptive exposure design, with 1 participant suggesting supplemental psychoeducation materials. Participants rated, on average, high satisfaction (≥4 on a 5-point Likert scale; 1=very dissatisfied and 5=very satisfied) with the interoceptive exposure (mean 4.3, SD 0.6), interoceptive counseling (mean 4.7, SD 0.6), and homework (4.0, SD 0.0) components of the RESET intervention.

### Exploratory Intervention Feasibility Results

Participants demonstrated pre-to-post changes in exercise sensitivity (baseline measurement: mean 28.0, SD 11.8; postintervention measurement: mean 33.7, SD 9.0; and pre- to postintervention change: mean 5.7, SD 7.5) and total physical activity levels (baseline measurement: mean 55.0, SD 56.8 min/wk; mean 888.2, SD 848.5 MET min/wk; postintervention measurement: mean 111.7, SD 7.6 min/wk; mean 2092, SD 611.1 MET min/wk; and pre-to postintervention change: mean 56.7, SD 63.3 min/wk; mean 1203.8, SD 1381.1 MET min/wk); however, statistical testing was not conducted.

## Discussion

### Principal Findings

This study provides a conceptual model and applied example of how to integrate IS principles and methods at earlier stages of behavioral intervention development in the form of a hybrid feasibility-implementation study. At stage I of behavioral intervention development, we simultaneously evaluated the intervention feasibility (adherence, fidelity, and outcome assessment) and implementation potential (feasibility, acceptability, and appropriateness) of a digital health–delivered behavioral intervention (ie, RESET) among midlife adult patients with ACS. According to our predefined criteria, we found that the RESET intervention was feasible but not implementable due to insufficient patient-perceived appropriateness, suggesting that we move back to stage 1A for further intervention refinement (ie, interoceptive exposure design). Our theory-informed, mixed methods approach provided breadth to our understanding of what aspects of RESET were not perceived as implementable and why as well as identified theory-informed contextual, behavioral, and innovation implementation determinants from the perspective of the innovation recipient (ie, patient), each of which will help inform efficient, reproducible refinements with the potential to enhance the effect and uptake of RESET at later stages of intervention development.

This is the first feasibility study to apply IS methods (ie, CFIR 2.0 and implementation outcomes proposed by Proctor et al [43]) to examine the implementation potential (ie, feasibility, acceptability, and appropriateness) of an early-stage behavioral health intervention in survivors of ACS. This approach enabled us to establish that it is feasible for patients with ACS to adhere to our intervention protocol and complete study assessments under controlled conditions [44] while also unveiling high patient-perceived implementation feasibility and acceptability of the intervention, 2 important perceptual implementation outcomes that have the potential to influence distal behavioral implementation outcomes in real-world settings (eg, adoption and penetration) [43,62]. We also found that our intervention protocol could be delivered with high fidelity and that the quality of intervention support and clarity of intervention instructions provided by the innovation deliverers are key facilitators to leverage for successful intervention implementation. Although promising, patients did not perceive the intervention as appropriate, primarily due to the lack of fit between the intervention design (ie, low interoceptive exposure dose) and intervention patient characteristics (ie, minimal fear of exercise), and key innovation barriers related to virtual delivery modality and poor intervention adaptability were identified. Interestingly, patients provided ample feedback on the design of the interoceptive exposure, with a specific focus on the lack of challenge provided by the walking activity, whereas little to no feedback was provided on the core intervention components designed to process sensations experienced during the interoceptive exposure (ie, interoceptive counseling and homework), suggesting that, to maximize efficiency of intervention feedback at earlier stages of intervention development, feasibility studies should consider enrolling a patient population that is an appropriate fit (vs convenience sample) for the proposed behavioral mechanisms and outcomes the intervention is intended to target.

A unique contribution of this study is the use of CFIR 2.0 to identify implementation determinants in combination with a mixed methods design feedback approach at stage I of behavioral intervention development, providing essential insights for an efficient and reproducible intervention refinement process. The CFIR 2.0 helped us identify contextual (available resources and access to knowledge and information), behavioral (capability, motivation, opportunity, and need), and innovation (design, adaptability, and complexity) determinants important for successful implementation, unveiling that most barriers to intervention uptake were linked to the innovation domain, while most facilitators were linked to the innovation recipient characteristics and inner setting domains. While these implementation determinants provide insights for how to adapt the intervention or create new materials to yield successful intervention implementation [57-60], linking satisfaction ratings and design feedback in a complementary fashion help inform which intervention design components to prioritize and why [53,63-65]. For example, we found that all patients disliked the interoceptive exposure design (due to low intensity, frequency, and duration) and expressed suboptimal satisfaction with the virtual delivery modality (due to poor visibility and usability of intervention materials on electronic devices), suggesting that we should focus on addressing barriers and leveraging facilitators relevant to these design elements as we revisit stage IA. This approach aligns with prior calls [23,66] and applied examples [63,67] for combining elements of user-centered design principles (ie, stakeholder engaged process to cocreate products that are directly responsive to the end user experience) with IS methods at early stages of intervention development and refinement; however, future studies are encouraged to engage multilevel stakeholders (eg, innovation deliverer and leaders) to more comprehensively inform real-world implementation potential. Although illustrative, our theory-informed, mixed methods approach can help intervention developers at all stages of intervention development identify what and understand why their intervention succeeded or failed to meet their implementation criteria, while providing theory-informed implementation determinants to guide how to approach intervention refinements or develop new materials (eg, education and implementation strategies).

### Strengths and Limitations

Our study has 2 key strengths. First, this study supplements the behavioral intervention development literature and responds to calls by experts in the field [16,23,26,27] by providing a conceptual model and applied example on the use of IS methods and principles at earlier stages of a well-established translational research framework (ie, NIH Stage Model). Our proposed “feasibility-implementation” study model and conceptual decision-making feasibility and implementation criteria may help intervention developers navigate earlier stages of intervention development according to key elements of the traditional translational research pipeline, while also taking into consideration key factors and design elements that influence implementation outcomes pertinent for real-world application (ie, designing with the end goal in mind). We focused on perceptual implementation outcomes (ie, acceptability and appropriateness), but other implementation outcomes (ie, cost, adoption, and penetration) may be relevant depending on the nature of the intervention and stage of intervention development [43,62]. Second, we are the first to use a theory-informed, mixed methods approach to identify and combine implementation determinants and design feedback at stage I of behavioral intervention development. This approach has the potential to aid intervention developers’ understanding of what, how, and why implementation efforts of an intervention succeed or fail [68,69], information that is useful during early and later stages of intervention development and can be applied to interventions as well as implementation strategies.

Our study findings should be interpreted in the context of several limitations. First, we had a small sample size of English-speaking, predominantly midlife adult patients from a single-center urban academic medical center, limiting the generalizability, depth, and inclusivity of our findings to other patients with ACS (eg, non–English-speaking, older adults) and implementation contexts. Due to the timing of our study (ie, during the COVID-19 pandemic), recruitment and study procedures took place remotely (requiring physician approval to approach patients), which negatively impacted our recruitment capabilities (Multimedia Appendix 4). Although recruitment was not considered an intervention feasibility outcome for our use case, as we focused primarily on intervention feasibility as opposed to trial feasibility (ie, pilot randomized study), we applied feedback and lessons learned among those contacted who declined to participate or were deemed ineligible to enhance recruitment efforts (Table S1 in Multimedia Appendix 2). We also documented barriers and facilitators to inform the development of recruitment strategies and materials needed for a future trial to be tested in a larger sample size inclusive of diverse older adult patient populations with ACS. Second, to be more inclusive and enhance recruitment success, we revised our inclusion criteria to have a lower exercise sensitivity and cardiac rehabilitation exposure threshold (Multimedia Appendix 2), which may have presented selection bias and influenced our findings. Accordingly, our enrolled patients had minimal exercise sensitivity and had already participated in a cardiac rehabilitation program, which may explain the high intervention feasibility and lack of appropriateness reported by the patients in this study. Third, intervention feedback from nonpatient stakeholders (eg, providers and health care system leaders) were not examined, hindering our ability to ensure our intervention embodies design elements responsive to the perspectives and experiences of other end users (ie, innovation deliverer) and decision makers (ie, leaders) who are essential to real-world implementation success. To address this limitation, we will engage in an iterative, multistakeholder, user-centered design, and IS-informed process to refine our intervention [67], an approach our group successfully used to develop a telehealth-delivered behavioral intervention [51]. Fourth, terminology (ie, feasibility-implementation study) and criteria established for our study were meant to be illustrative and were informed by the literature and our team’s health-related behavioral intervention development and IS expertise. Given the overlap in terminology (ie, feasibility and fidelity) and frameworks (ie, refinement and adaptation) used in the fields of behavioral intervention development and IS, future studies are needed (ie, Delphi polling and concept mapping) to establish evidence-based consensus from experts in both fields on relevant terminology and decision-making criteria to be applied at earlier stages of intervention development [63]. Fifth, our conceptual model is not applicable to all early-stage behavioral interventions, as some interventions (eg, high-risk exposure, behavior, or outcome) should not consider integrating IS principles and methods until further pilot and efficacy testing has been conducted. Finally, we acknowledge that our proposed approach is time and resource intensive and may not be feasible at earlier stages of intervention development. Despite these limitations, our findings shed light on how to integrate methods, outcomes, and principles underpinning the field of IS into earlier stages of behavioral intervention development.

### Conclusions

This paper provides a conceptual model and applied example for integrating IS methods, outcomes, and principles into the early-stage development of behavioral interventions. We found that evaluating intervention feasibility and implementation potential simultaneously may help intervention developers navigate earlier stages of intervention development according to key elements of the traditional translational research pipeline, while also taking into consideration key factors that influence real-world implementation. We also found that a theory-informed, mixed methods approach can elucidate what, how, and why implementation efforts of an intervention succeed or fail, information that is useful during early and later stages of intervention development. Our proposed feasibility-implementation study design may serve as a useful model for intervention developers that aim to address implementation issues as early as possible in the intervention development process, while ensuring the intervention reaches its maximum effectiveness in real-world settings.

## Appendix

Multimedia Appendix 1 Preintervention preparation materials and instructions.

Multimedia Appendix 2 Supplemental intervention methods, protocol changes, interoceptive counseling data collection sheet, homework materials, and semistructured interview guide.

Multimedia Appendix 3 Psychoeducation presentation materials.

Multimedia Appendix 4 CONSORT (Consolidated Standards of Reporting Trials) diagram, implementation outcome ratings, intervention design feedback, and intervention satisfaction ratings.

Multimedia Appendix 5 Implementation determinant themes, codes, and representative quotes categorized by the Consolidated Framework of Implementation Research 2.0 domains and constructs.

## Abbreviations

ACS: acute coronary syndrome
AIM: Acceptability of Intervention Measure
CFIR: Consolidated Framework for Implementation Research
CONSORT: Consolidated Standards of Reporting Trials
COREQ: Consolidated Criteria for Reporting Qualitative Studies
CUIMC: Columbia University Irving Medical Center
CVD: cardiovascular disease
ESQ-18: 18-item Exercise Sensations Questionnaire
FIM: Feasibility of Intervention Measure
G6MW: gradual 6-minute walk
IAM: Intervention Appropriateness Measure
IPAQ-SF: International Physical Activity Questionnaire-Short Form
IS: implementation science
MET: metabolic equivalent task
NIH: National Institutes of Health
RESET: Reducing Exercise Sensitivity with Exposure Training

## Data Availability

The datasets generated and analyzed during this study are not publicly available to protect participant anonymity but are available from the corresponding author on reasonable request.
