# Supplementary material for: Leveraging Implementation Science at the Early-Stage Development of a Novel Telehealth-Delivered Fear of Exercise Program to Understand Intervention Feasibility and Implementation Potential: Feasibility Behavioral Intervention Study
Source: JMIR Form Res. 2024 Nov 12;8:e55137. doi: 10.2196/55137 (PMC11599889; doi:10.2196/55137)
Supplement: Multimedia Appendix 1 [file formative_v8i1e55137_app1.pdf]

reset

Reducing Exercise
Sensitivity with
Exposure Training

Welcome!

reset Intervention Design

Intervention Prep
(60-90 mins)

Open RESET Kit:
Fitbit, Agility
Cones, Measuring
Tape

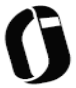

Fitbit Inspire
HR

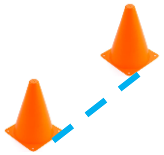

Walking
Course

Video Visit 2
(60 mins)

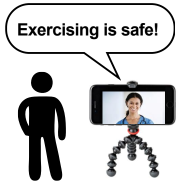

Education

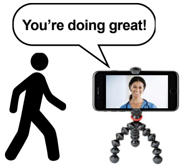

Walking Activity

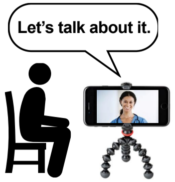

Counseling and
Questionnaires

Video Visit 3
(30 mins)

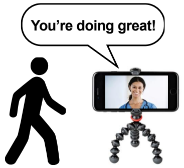

Walking Activity

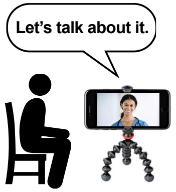

Counseling

Post-Intervention
(30-45 mins)

Questionnaires and
Exit Interview

reset
is a remote
home-based
intervention

zoom
Video Communications

RESET journal Participant documents physical activity and exercise sensations

Materials

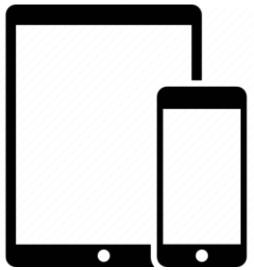

Tablet or Smartphone
(iPhone or Android)

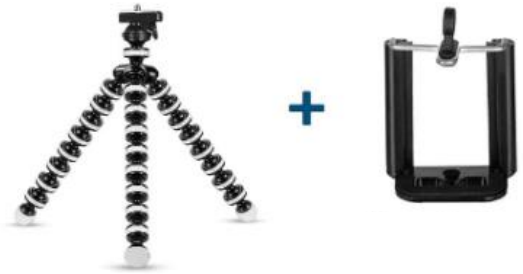

Tripod Stand

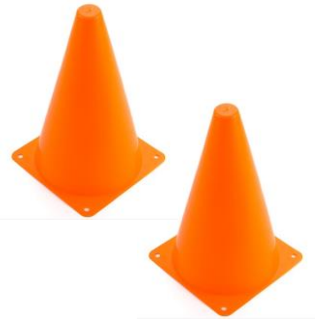

Agility Cones

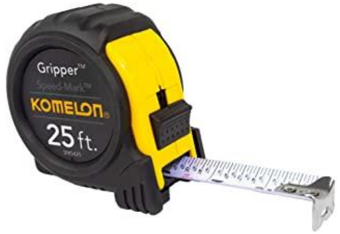

Tape Measure

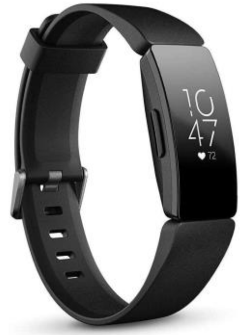

Fitbit Inspire HR

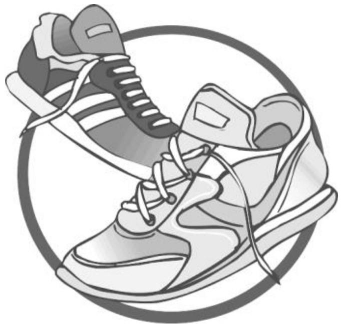

Comfortable clothes
and walking shoes

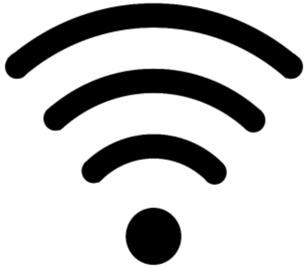

Internet

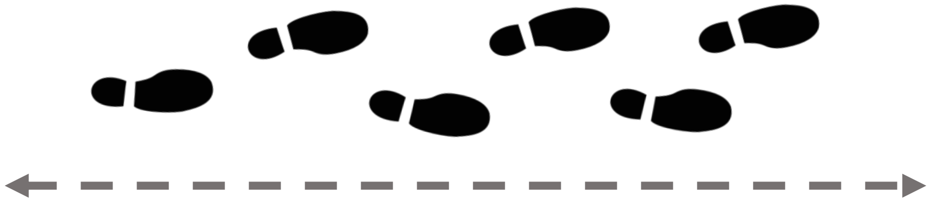

Flat floor space

Step 1

Open the box that arrived in the mail and ensure all materials are present.
Make sure you are wearing comfortable walking shoes and clothing!

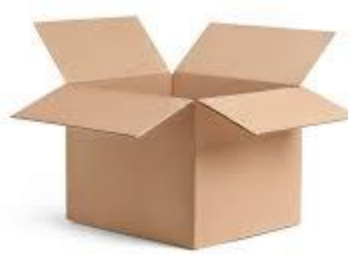

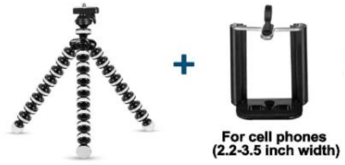

Tripod Stand

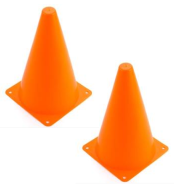

Agility Cones

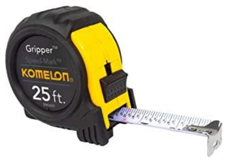

Tape Measure

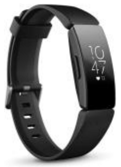

Fitbit Inspire HR

Step 2

Find a flat, open floor space in your home. Try to remove any obstacles that might get in your way. Use the tape measure included in your kit to measure about 6 feet and place the tape measure on the floor. Place a cone at each end of the tape measure. This is your walking course!

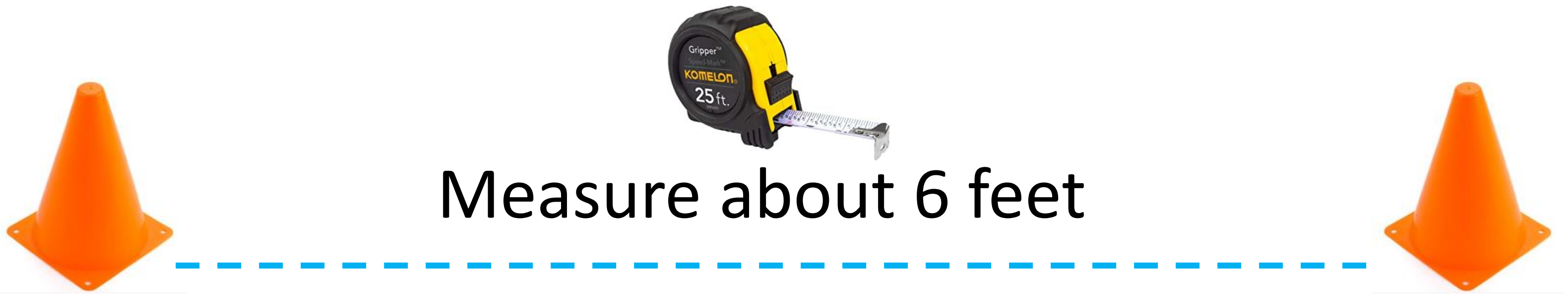

Step 3

Assemble the gorilla pod/tripod stand and place your phone horizontally into the phone holder so that it is secure.

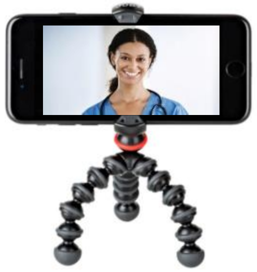

Step 4

Set up the gorilla pod so that the research personnel can view you and the full length of the walking course during the session. Your face should show up on video when you are walking towards your phone. Your walking coach can help you make sure your camera is set up properly and they can see you clearly.

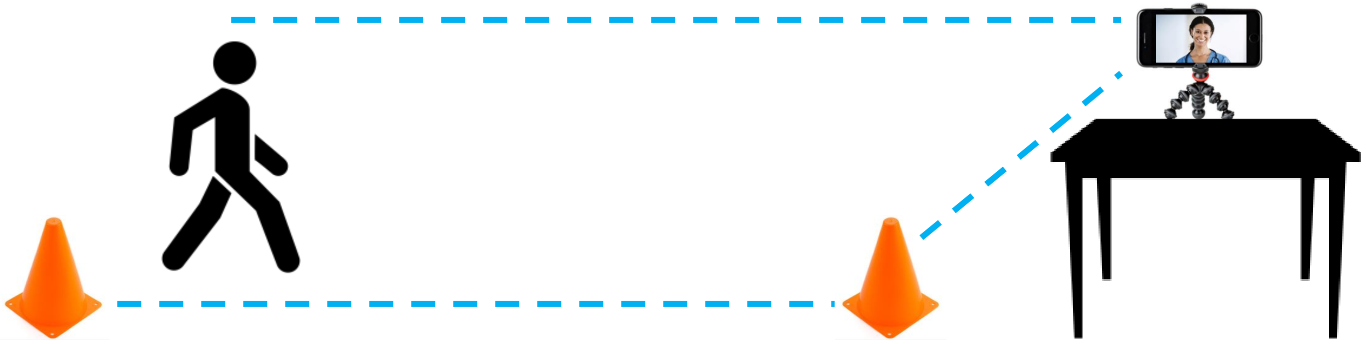

Walking Activity Overview

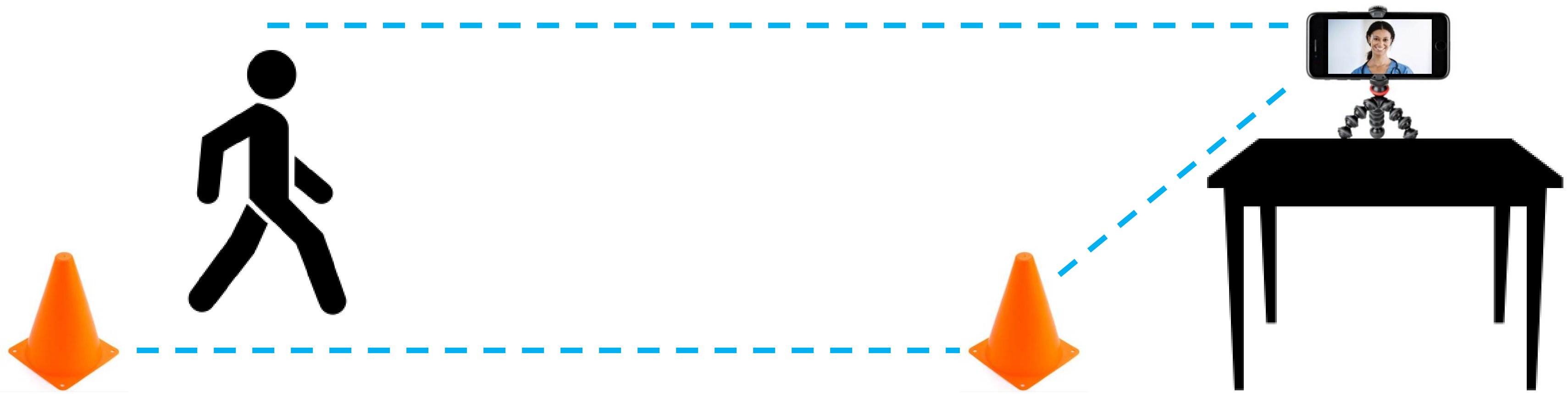

6 Minute Walking Activity

For the first minute we'll start slowly – about 50 steps per minute.
We'll try and go a little bit faster each minute.
For the last minute we'll try to walk at a moderate pace- about 100 steps per minute.

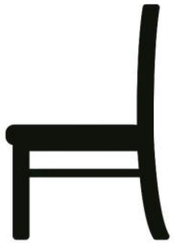

Rest

When you finish the walking exercise, sit in chair for 3-5 minutes and take recovery heart rate and perceived exertion

reset

Reducing Exercise Sensitivity with Exposure Training

Let’s get started!
