# Supplementary material for: Leveraging Implementation Science at the Early-Stage Development of a Novel Telehealth-Delivered Fear of Exercise Program to Understand Intervention Feasibility and Implementation Potential: Feasibility Behavioral Intervention Study
Source: JMIR Form Res. 2024 Nov 12;8:e55137. doi: 10.2196/55137 (PMC11599889; doi:10.2196/55137)
Supplement: Multimedia Appendix 2 [file formative_v8i1e55137_app2.docx]

**Multimedia Appendix 2**

# **Title:** Leveraging Implementation Science at the Early-Stage Development of a Novel Telehealth-Delivered Fear of Exercise Program to Understand Intervention Feasibility and Implementation Potential: Feasibility Behavioral Intervention Study

**Authors:**

Andrea T. Duran^1^, MPhil, PhD; Robin M. Cumella^1^, BA; Miguel Mendieta^1^, BA; Adrianna Keener-Denoia^1^, MA; David López Veneros^1,2^, RN, MA, MPhil; Samantha G. Farris^3^, PhD; Nathalie Moise^1^, MS, MD; Ian M. Kronish^1^, MPH, MD

^1^Center for Behavioral Cardiovascular Health, Columbia University Irving Medical Center, New York, NY, United States

^2^Columbia University School of Nursing, New York, NY, United States

^3^Department of Psychology, Rutgers, The State University of New Jersey, Piscataway, NJ, United States

**Corresponding Author:**

Andrea T Duran, MPhil, PhD

Center for Behavioral Cardiovascular Health

Columbia University Irving Medical Center

622 West 168th Street

New York, NY, 10032

United States

Phone: 1 212 342 4491

Email: [atd2127@cumc.columbia.edu](mailto:atd2127@cumc.columbia.edu)

**Supplemental Intervention Methods.**

**RESET Intervention:** A member of the research team met with the participant on a secure Zoom Video Visit platform to conduct a total of 2 RESET intervention video visits. Each RESET intervention video visit occurred once or twice per week based on participant preference. The first intervention visit (Video Visit 1) included psychoeducation, interoceptive exposure (i.e., G6MW), and interoceptive counseling. At the end of Video Visit 1, participants completed a series of valid, reliable, and pragmatic 4-item surveys designed to assess the acceptability, appropriateness, and feasibility of the RESET program, as well as the satisfaction of each intervention component and design feedback (e.g., psychoeducation, interoceptive exposure, interoceptive counseling [1]. The other intervention visit (Video Visit 2) included interoceptive exposure (i.e., G6MW) and interoceptive counseling. At the end of the last intervention visit (Video Visit 2), participants completed post-intervention questionnaires and an exit interview. Details of each RESET intervention component are provided below.

***Psychoeducation (30 mins):*** Content included in the psychoeducation built off core elements from cognitive-behavioral models of anxiety. The research team member started by reviewing participants’ specific feared sensations endorsed on the ESQ and the cognitive-behavioral responses to feared sensations. Then, participants were provided with psychoeducation on the following main topics: (1) exercise and cardiac rehabilitation, (2) exercise sensitivity, and (3) interoceptive exposure. For the first topic, participants learned about the importance, benefits, and safety of exercise and cardiac rehab participation for heart health, as well as the fundamentals (e.g., frequency, intensity, time, type) to engaging in a safe home-based exercise routine. For the second topic, participants learned about the biological basis of fear (our body’s alarm system) and normal physical sensations that are healthy, but often feared during exercise. For the third topic, participants learned about exercise fears-avoidance as a barrier to exercise and CR, common exercise avoidance behaviors (e.g., slowing down, discontinuation of exercise) that can occur in the presence of aversive bodily sensations, and the concept of interoceptive exposure practice - the use of approach behavior (vs avoidance) - to decrease fear and build tolerance to anxiety and exercise. At the end of the psychoeducation session, the research team collaboratively problem-solved any potential barriers to engaging with or attending treatment (e.g., technological concerns of using telemedicine delivery). The patient-facing Power Point presentation used to guide the psychoeducation session is provided in Multimedia Appendix 3.

***Interoceptive exposure (15 mins):*** Interoceptive exposure is one component of cognitive-behavioral therapy that repeatedly engages participants in a series of exercises (e.g., structured physical activity) that provoke feared physical sensations [2]. For this intervention, a gradual six-minute walk (G6MW) (i.e., six-minute bout of light-to-moderate intensity physical activity) served as a low-risk form of interoceptive exposure. To ensure our participants engaged in a walking bout that did not exceed moderate-intensity exercise, participants were instructed to walk at a cadence between 50 (light intensity) and 100 steps/min (moderate intensity). Participants were asked to slow down if they exceeded 100 steps/min. This step cadence was based on the findings from Tudor-Locke and Colleagues (2020) [3], as well as aligns with the standard of care physical activity guidelines for secondary prevention [4]. Stepping cadence was remotely monitored and regulated with a metronome. If the participant’s home-environment was not conducive for proper monitoring of the walking activity, or they were not uncomfortable displaying their home environment, they were given the opportunity to complete the physical activity session by walking in place.

Prior to the G6MW, participants were provided with rationale and instructions for the walking exposure. Then, the research team member asked the following questions: (1) “What sensations do you expect to feel while you are walking?”; (2) “How intense do you expect this sensation to be?”, (3) “How distressing do you expect this sensation to be?”. Questions 2 and 3 were rated for each sensation reported in Question 1 on a 0-10 scale (where 0=not at all distressing/tolerable, and 10=extremely distressing/tolerable). Next, the research team member explained the Borg Category-Ratio 10 (CR10) scale, which was used to measure the participant’s rating of perceived exertion (RPE) during physical activity (0=nothing at all, and 10=extremely strong [“maximal”]) [5]. Once all instructions and questions were administered, the research team member started the metronome (50 bpm) and instructed the participant to start the walking exposure (e.g., G6MW). Throughout the G6MW, the research team member increased the cadence of the metronome by 10 bpm (maximum cadence: 100 bpm) and measured exertion levels using the Borg CR10 scale at 1-minute intervals. We also evaluated the extent to which participants titrated the intensity of exercise (i.e., slowed down) or discontinued in the presence of fears about bodily sensations. Immediately after the G6MW, participants were instructed to sit down in a chair and rest for 3-5 minutes. Heart rate was measured before (e.g., rest) and after (e.g., recovery) the G6MW.

***Interoceptive Counseling (10-20 mins):*** After the participant was rested, the research team member asked the following questions: (1) “What sensations did you experience while you were walking?”, (2) “How intense was this sensation?”, (3) “How distressing was this sensation?”, (4) “During the walking activity, did you have an urge to slow down or stop?”. Questions 2 and 3 were rated for each sensation reported in Question 1 on a 0-10 scale (where 0=not at all distressing/tolerable, and 10=extremely distressing/tolerable). Next, the research team member shared the pre-walking activity and post-walking activity data sheets on the Zoom screen (see **Figure S1**) and spent time reviewing the pre-walking exposure and post-walking exposure ratings with the participant to help facilitate the feedback and processing of the exposure. The goal of comparing pre- vs. post-walking exposure ratings was to identify which aspects of the walking exposure participants perceived as *surprising* (e.g., “It was not as bad as I expected”), *different* (e.g., “I thought I would feel X, but I actually felt Y”), or *similar* (e.g., “It was just as I expected… uncomfortable”) to their initial expectations about exercise. Each of these perceptions (e.g., surprising, different, similar) provided participants with feedback that their initial expectations about exercise may or may not have been fully accurate, and either way, tolerating and experiencing distressing sensations can help them be prepared to make exercise a part of their lifestyle and/or exercise in cardiac rehab in the future. After counseling was completed, research personnel worked with the participant to develop a comfortable stepping cadence progression for their next walking exposure session.

***Homework:*** Participants were encouraged (but not required) to achieve ≥30 minutes of moderate aerobic activity, such as brisk walking, on ≥5 days per week. Participants were instructed to document their habitual physical activity (i.e., date, time of day, duration, modality) and exercise sensations in a weekly journal, to help participants reflect on their physical activity experiences. To familiarize participants with the journal, a member of the research team spent ~5 mins reviewing the weekly journal template at the end of video visit 1 (see **Figure S2**). To do so, an electronic version of the journal was shared on the Zoom video visit screen and a member of the research team verbally and visually reviewed the journal instructions and each journal entry field (e.g., What sensations did you experience during physical activity?). To ensure participant comprehension, examples for each journal entry field were provided (e.g., rapid heart rate, shortness of breath). Participants were asked to use the journal for either one or two weeks from the start of the intervention (i.e., Video Visit 1) in direct correlation with their self-selected frequency of intervention visits (i.e., once a week or twice a week). Physical activity and journal entries were reviewed with the research team member at the final intervention visit (i.e., Video Visit 2). At the end of the intervention, participants mailed back their journal in a pre-paid package.

**Table S1. Overview of major study protocol changes and rationale for changes.**

| **Study Protocol Change** | **Rationale** |
| --- | --- |
| Reduced the total number of Zoom-delivered intervention sessions from four to two. | The initial study design included a total of four intervention study visits, which participants could choose to schedule either once or twice a week. However, after several patients declined to participate in the study, citing that they were too busy to participate, the study team reduced the number of intervention study visits to two (which could also be scheduled once or twice a week). |
| Removed CR attendance as an exclusion criteria. | Cardiac rehabilitation (CR) is a comprehensive, multi-component, evidence-based secondary prevention program that includes patient assessment, exercise training, and educational counseling (nutrition, psychosocial, physical activity, risk factor management). Given that a CR program provides overlapping, yet more intense, intervention components (i.e., exercise, education) as our intervention, the initial study design excluded any ACS patient who had participated in more than one cardiac rehabilitation session as ineligible, as we hypothesized that our intervention would be most appropriate for ACS patients who had not participated in a CR program. We decided to remove this exclusion criterion in an effort to be more inclusive of any ACS patients within 12 months of their event who still endorsed some fear of exercise, as well as learn if this intervention could be helpful in this population. |
| Added ESQ18 to inclusion criteria. | The initial screening protocol required patients to score >1 (*sometimes*, *often*, or *very often*) on at least one item from the four-item Aversive Cognitions about Physical Activity Scale as method to detect fear of exercise [6]. This pragmatic scale was chosen based on its successful use in ACS patients from our prior studies and as a means to reduce participant burden. However, after several participants who consented to the study screened ineligible based on their responses to the Aversive Cognitions about Physical Activity Scale, and participant feedback that the screening questions were too short, we wanted to ensure that the screener for the study was more comprehensive and sensitive enough to detect some fear of exercise. Therefore, we added the 18-item Exercise Sensations Questionnaire (ESQ-18) as an additional measure to detect fear of exercise, as it has been validated in a cardiac patient population and may be more sensitive to detecting fear of the sensations experienced with exercise [7]. Accordingly, to meet our fear of exercise inclusion criterion, score >1 (*sometimes*, *often*, or *very often*) on at least one item from the four-item Aversive Cognitions about Physical Activity Scale and/or score >1 (*some, much, or very much*) on at least one item from the 18-items Exercise Sensations Questionnaire (ESQ-18). |

**Figure S1.** Example of the pre-walking activity and post-walking activity data sheets and potential participant responses.

**
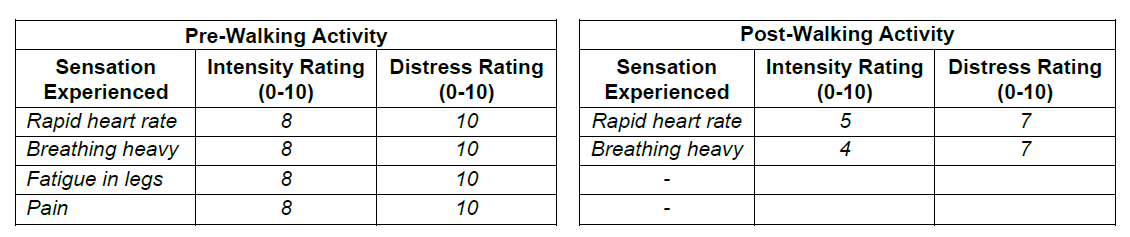
**

**Figure S2.** Example of weekly journal provided to participants as homework.


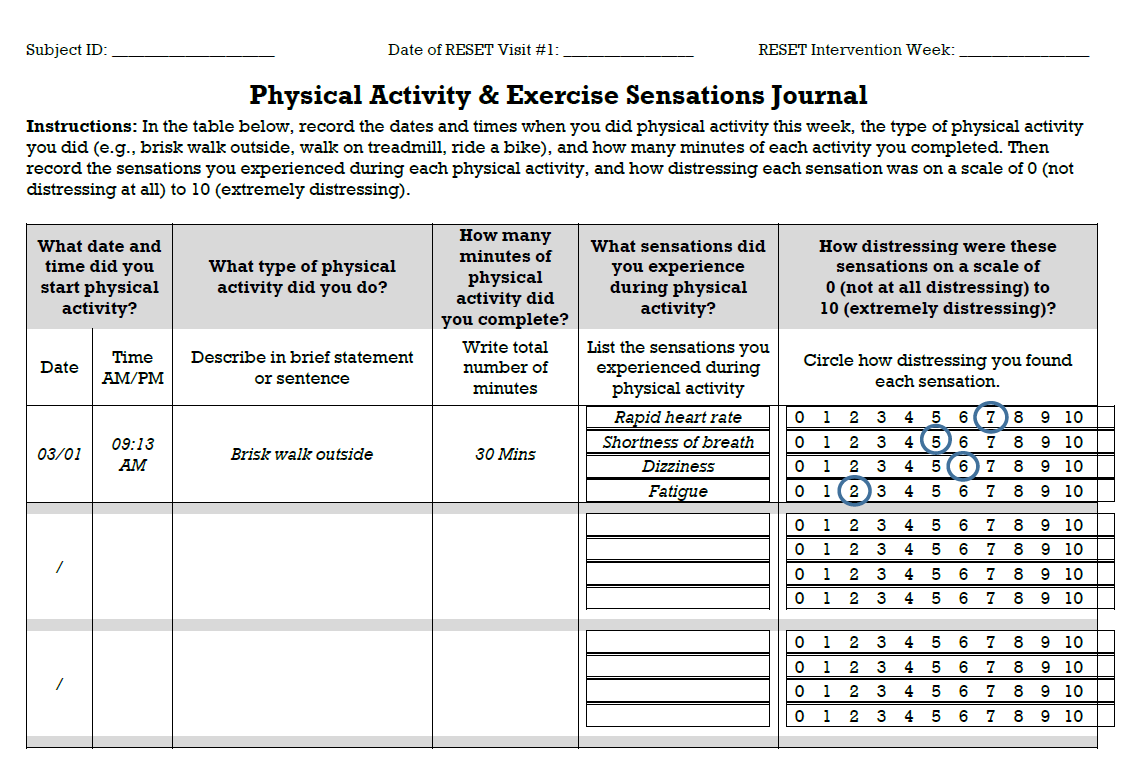


**Semi-structured interview guide questions.**

**Q1. In general, how would you describe your experience with participating in the RESET program?**

Q1a. Overall, would you describe your experience as enjoyable? Why or why not?

Q1b. What were your thoughts about the total time commitment (e.g., ~3-4 weeks)? Was it too much, too little, or about right?

Q1c. What were your thoughts on the total number of video visits (e.g., 3 video visits)? Would you prefer more/less video visits?

Q1d. How well did the RESET team members communicate (e.g., clearly,effectively? How could their communication be improved?

**Q2. What were the *challenges or concerns* you experienced while participating in the RESET program?**

*Q2a. To what extent did you experience specific challenges or concerns while participating in the:*

- *educational session?*
- *gradual six-minute walking activity?*
- *counseling session?*
- *homework?*

**Q3. What would have *helped* you participate to the fullest extent in the RESET program?**

**Q4. What did you like the *most* about the RESET program?**

**Q5. What did you like the *least* about the RESET program?**

**Q6. What would you *change* about the RESET program?**

Prompts:

- educational session
- gradual six-minute walking activity
- counseling session
- homework

**Q7. If a family member or colleague were to consider participating in the RESET study, what would you tell them? Why?**

**Q8. Do you have anything else you would like to add about your experience with the RESET program?**

**Q9. Do you have anything else you would like to add about how to improve the RESET program design?**

**References**

1. Weiner BJ, Lewis CC, Stanick C, Powell BJ, Dorsey CN, Clary AS, et al. Psychometric assessment of three newly developed implementation outcome measures. Implement Sci. 2017 Aug 29;12(1):108. PMID: 28851459. doi: 10.1186/s13012-017-0635-3.

2. Holtz K, Hamm AO, Pane-Farre CA. Repeated Interoceptive Exposure in Individuals With High and Low Anxiety Sensitivity. Behav Modif. 2019 Jul;43(4):467-89. PMID: 29690770. doi: 10.1177/0145445518772269.

3. Tudor-Locke C, Ducharme SW, Aguiar EJ, Schuna JM, Jr., Barreira TV, Moore CC, et al. Walking cadence (steps/min) and intensity in 41 to 60-year-old adults: the CADENCE-adults study. Int J Behav Nutr Phys Act. 2020 Nov 10;17(1):137. PMID: 33168018. doi: 10.1186/s12966-020-01045-z.

4. Anderson JL, Adams CD, Antman EM, Bridges CR, Califf RM, Casey DE, Jr., et al. 2011 ACCF/AHA Focused Update Incorporated Into the ACC/AHA 2007 Guidelines for the Management of Patients With Unstable Angina/Non-ST-Elevation Myocardial Infarction: a report of the American College of Cardiology Foundation/American Heart Association Task Force on Practice Guidelines. Circulation. 2011 May 10;123(18):e426-579. PMID: 21444888. doi: 10.1161/CIR.0b013e318212bb8b.

5. Borg GA. Psychophysical bases of perceived exertion. Med Sci Sports Exerc. 1982;14(5):377-81. PMID: 7154893.

6. Monane R, Sanchez GJ, Kronish IM, Edmondson D, Diaz KM. Post-traumatic stress disorder symptoms and aversive cognitions regarding physical activity in patients evaluated for acute coronary syndrome. Eur J Prev Cardiol. 2018 Mar;25(4):402-3. PMID: 29198138. doi: 10.1177/2047487317746255.

7. Farris SG, Abrantes AM, Bond DS, Stabile LM, Wu WC. Anxiety and Fear of Exercise in Cardiopulmonary Rehabilitation: PATIENT AND PRACTITIONER PERSPECTIVES. Journal of cardiopulmonary rehabilitation and prevention. 2019 Mar;39(2):E9-E13. PMID: 30801438. doi: 10.1097/HCR.0000000000000401.
