# Supplementary material for: Leveraging Implementation Science at the Early-Stage Development of a Novel Telehealth-Delivered Fear of Exercise Program to Understand Intervention Feasibility and Implementation Potential: Feasibility Behavioral Intervention Study
Source: JMIR Form Res. 2024 Nov 12;8:e55137. doi: 10.2196/55137 (PMC11599889; doi:10.2196/55137)
Supplement: Multimedia Appendix 3 [file formative_v8i1e55137_app3.pdf]

reset

Reducing Exercise Sensitivity with Exposure Training

Welcome!

Background

**Exercise concerns among patients with heart disease**	**Percent**
Not being able to breathe	42%
Having a cardiac event	45%
Stop or slow down due to bodily sensations	72%
Beneficial to address fears of exercise in cardiac rehab	75%

Frequently Asked Questions

Why should I exercise?

Why should I attend cardiac rehabilitation?

Is it safe for me to exercise?

Is it safe for me to exercise without supervision?

What are the normal responses to exercise?

Benefits of Exercise and Cardiac Rehab

Lower risk of having another heart attack or heart-related medical event

Lower risk of developing other health conditions

Lower risk of mortality (death)

Improve healthy weight management

Improve muscle, bone, and joint health

Improve brain, mental, and emotional health

Improve quality of life and overall well-being

Exercise Fundamentals

Intensity: How hard you exercise

Vigorous: Fast running, hiking uphill, heavy yardwork

Moderate: Brisk walking, dancing, gardening

Light: Slow walking, house-hold chores (vacuuming, mopping)

Frequency: How often you exercise (days/week)

Duration: How long you exercise (mins per exercise session)

Recommendation:
Exercise at light to moderate intensity for ≥ 30 minutes on ≥ 5 days per week

What are normal exercise responses?

Normal Exercise Response

Heart beating faster

Heart pounding

Shortness of breath—able to talk but not sing

Fatigue in your leg muscles

Feeling tired from working hard

Growing flushed with exertion

Stuck in the Cycle

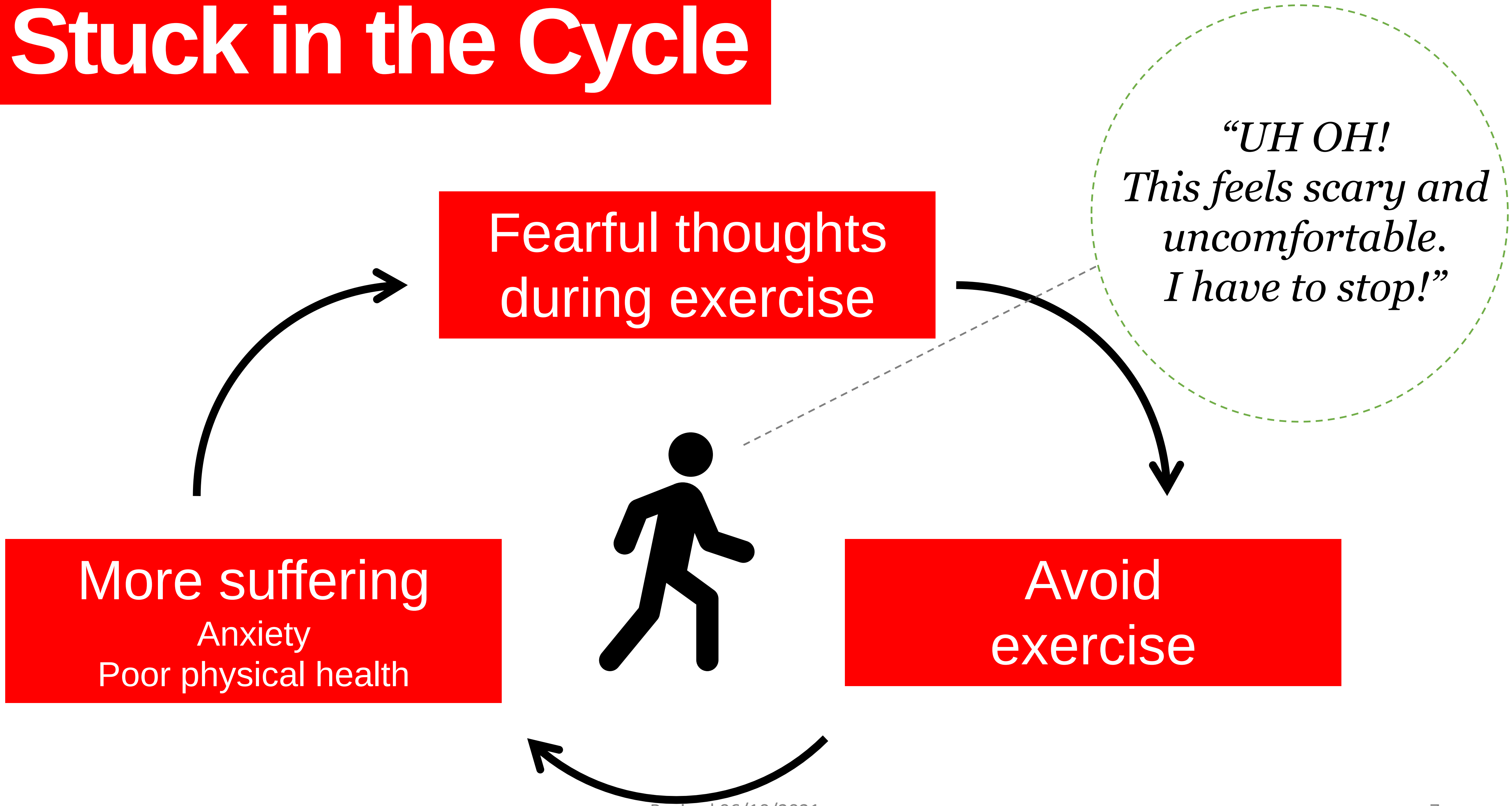

Breaking the Cycle

Fearful thoughts during exercise

"I can accept this feeling.

I'm okay!"

Approach exercise

Embracing a physically active lifestyle

The cycle is broken!

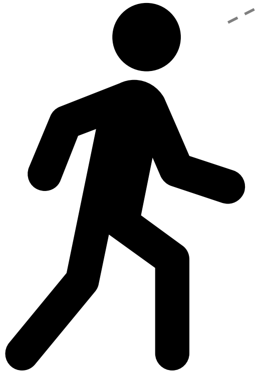

reset

Reducing Exercise Sensitivity with Exposure Training

Let's get started!
