# Supplementary material for: Leveraging Implementation Science at the Early-Stage Development of a Novel Telehealth-Delivered Fear of Exercise Program to Understand Intervention Feasibility and Implementation Potential: Feasibility Behavioral Intervention Study
Source: JMIR Form Res. 2024 Nov 12;8:e55137. doi: 10.2196/55137 (PMC11599889; doi:10.2196/55137)
Supplement: Multimedia Appendix 4 [file formative_v8i1e55137_app4.docx]

**Multimedia Appendix 4**

# **Title:** Leveraging Implementation Science at the Early-Stage Development of a Novel Telehealth-Delivered Fear of Exercise Program to Understand Intervention Feasibility and Implementation Potential: Feasibility Behavioral Intervention Study

**Authors:**

Andrea T. Duran^1^, MPhil, PhD; Robin M. Cumella^1^, BA; Miguel Mendieta^1^, BA; Adrianna Keener-Denoia^1^, MA; David López Veneros^1,2^, RN, MA, MPhil; Samantha G. Farris^3^, PhD; Nathalie Moise^1^, MS, MD; Ian M. Kronish^1^, MPH, MD

^1^Center for Behavioral Cardiovascular Health, Columbia University Irving Medical Center, New York, NY, United States

^2^Columbia University School of Nursing, New York, NY, United States

^3^Department of Psychology, Rutgers, The State University of New Jersey, Piscataway, NJ, United States

**Corresponding Author:**

Andrea T Duran, MPhil, PhD

Center for Behavioral Cardiovascular Health

Columbia University Irving Medical Center

622 West 168th Street

New York, NY, 10032

United States

Phone: 1 212 342 4491

Email: [atd2127@cumc.columbia.edu](mailto:atd2127@cumc.columbia.edu)

**Figure S1.** Study Consort Flow Diagram


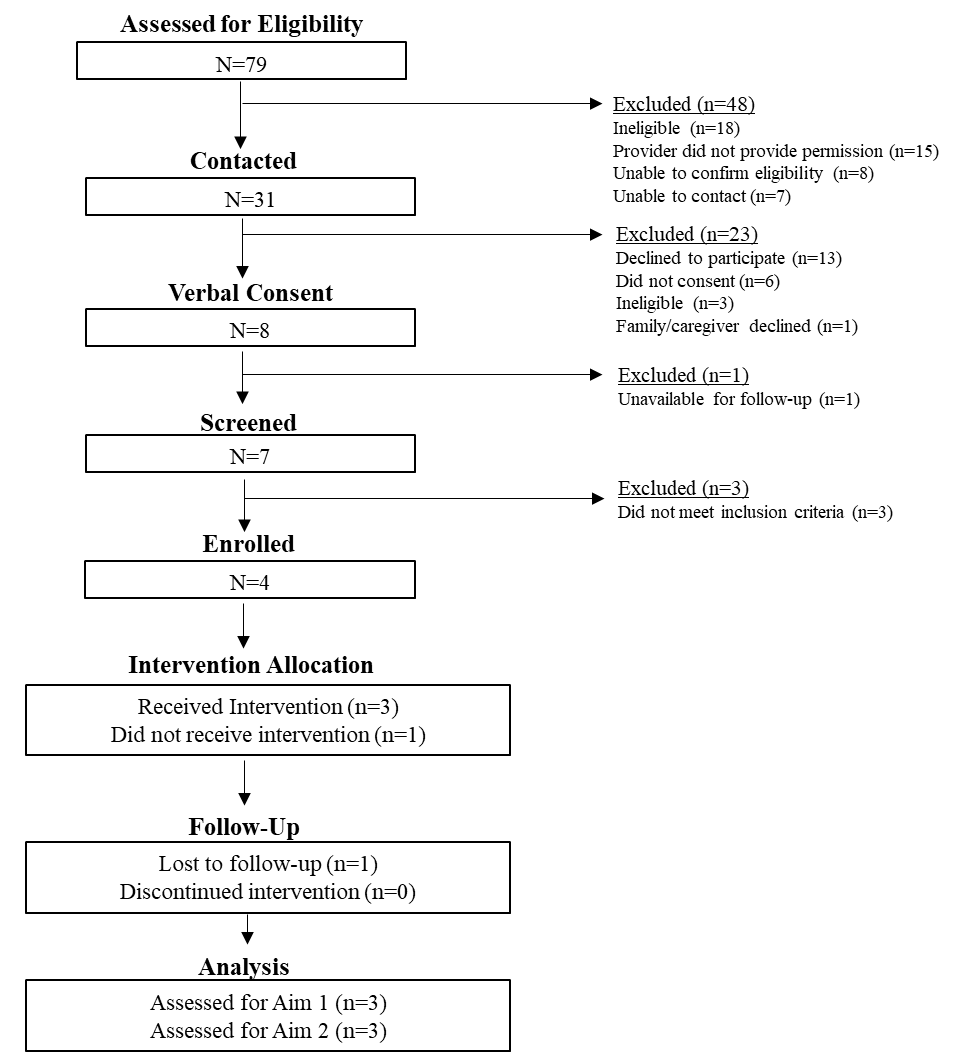


Table S1. Participant feedback and implementation outcome survey ratings mapped onto intervention feasibility, acceptability, and appropriateness.

| **Implementation Outcome** (definition) | **Survey Rating** (mean±SD) | **Representative Quotes** |
| --- | --- | --- |
| **Intervention Feasibility** (intervention can be used or carried out within a given agency or setting) | 4.2±0.4 | “The challenge that I had, is more of a, you know, the space issue for setting up the course. […] Because, I think, for me (the intervention walking course) was a little bit short...a couple of steps and a couple of steps." ID_451  “So, it was hard to just set (the intervention walking course) up on a, you know, just on the carpet. In a normal situation, if I don't take into consideration that uneven surface it would not be a problem." ID_451  "I thought it was hard to see some of the prompts and it appeared that it was like kind of unclear to see...on the screen. And then because the phone, I used on my phone and it was like kind of small." ID_439  “It's difficult to adjust everything and keep the walking course in view. Hard to find the right angles.” ID_439  "What I liked is that it is convenient because you can do it on your own schedule...And you can do it from home." ID_451  "It makes it a lot easier when you have the space to do your exercise at home. It's more convenient, more comfortable. " ID_449 |
| **Intervention Acceptability** (intervention is agreeable, palatable, or satisfactory) | 4.3±0.7 | "The homework was easy, you know, write down how you feel. That's it. That's easy.” ID_449  "(The intervention) wasn't too time-consuming either… It wasn't like excessive." ID_439  "I'm satisfied with the experience. I think (the intervention is) a good program." ID_451  "Study equipment was ... useful and I got everything in a timely manner, all the materials received. And everything worked, so that was good. " ID_439  "(The intervention) was wonderful and interesting. I learned a lot." ID_449 |
| **Intervention Appropriateness** (perceived fit, relevance, or compatibility of the intervention for a given patient, and/or a particular issue) | 3.7±0.4 | "(Patients) got so used to just sitting here, laying in the bed, laying in the bed, and not moving ... Those are the people that really, really do need real help." ID 449  “I think (the intervention is) the first step forward after a heart attack.” ID_451  "…If you were in the beginning stages or it was a more recent cardiac event, I think (the intervention) would be more appropriate for someone who is somewhat reluctant." ID_439  “(The intervention is) not for me. I would say it's for other participants. … the basic two sessions what we did for the walking exercise, I think it gives (other patients) some confidence, but probably for some people it doesn't give enough….maybe a little bit longer, more, more walking sessions involved (for patients who) were close to their event...but you know, it's scary." ID_451  "The walking part, I think was a little slow for me. It was a little slow. I could have walked a little bit faster… The pace part is the only thing I would change." ID_449 |

Table S2. Participant satisfaction and proposed design changes for each RESET intervention component and delivery modality.

| **Variable** | **Satisfaction Rating**  (mean±SD) | **Design Feedback** | **Representative Quotes** |
| --- | --- | --- | --- |
| **Intervention Component** |  |  |  |
| Psychoeducation | 3.7 (0.6) | Supplemental Materials | “What would have helped participate to the fullest extent? Study materials or some YouTube video that tries to explain the situation better for the people so they can get more comfortable with that...which can explain them the facts better." ID_451 |
| Interoceptive Exposure | 4.3 (0.6) | Increase/Tailor Intensity | “Maybe like an option for like a higher level intervention of increased challenge is what I'd say. Like having the option is what would be a good thing is what I would add.” ID_439  “We do the pace according to each individual person. Some people can walk slow, some people can walk medium, some people can walk fast, some people can walk [inaudible]. I would say, according to the person's pace, it may help.” ID_449  “Make (the pace) individual to each client. What they can do and what they can’t do.” ID_449 |
|  |  | Increase Duration and Frequency | "I guess (the interoceptive exposure activity) could have been a little longer. Like duration slash frequency…Once you got pumped up it just kind of stopped, you know what I mean?" ID_439  "Maybe a little bit longer, more, more walking sessions involved for (people who) were close to their event." ID_451  "And then, I guess, well, it really isn't that long. Like 6 minutes and whatnot. So it could have been longer, I could have, I could have done it longer." ID_439 |
| Interoceptive Counseling | 4.7 (0.6) | None | “No challenges. No concern.” ID_449 |
| Homework | 4.0 (0.0) | None | "(The homework was) pretty straightforward." ID_439  "The homework was easy, you know, write down how you feel. That's it. That's easy.” ID_449 |
| **Intervention Delivery Modality** |  |  |  |
| Ease of seeing intervention materials on electronic device during video visits | 3.3 (1.5) | Alternative electronic devices/Bigger Screen | “Setting up for the walking activity would be easier on a bigger screen.” ID_439  "I thought it was hard to see some of the prompts and it appeared that it was like kind of unclear to see...on the screen. And then because the phone, I used on my phone and it was like kind of small." ID_439  “It's difficult to adjust everything and keep the walking course in view. Hard to find the right angles.” ID_439 |
| Ease of hearing and seeing interventionist on electronic device during video visits | 4.3 (0.6) | None | "(The study team) communication was great. They talked slow. Clearly. If I didn't understand something, they would explain it to me so that I can understand it...They did a great job explaining exactly what entitled, what is expected, what is not expected. It was great. I had no complaints." ID_449  “I don't have any. I think the communication was good.” ID_451 |
