# Supplementary material for: Leveraging Implementation Science at the Early-Stage Development of a Novel Telehealth-Delivered Fear of Exercise Program to Understand Intervention Feasibility and Implementation Potential: Feasibility Behavioral Intervention Study
Source: JMIR Form Res. 2024 Nov 12;8:e55137. doi: 10.2196/55137 (PMC11599889; doi:10.2196/55137)
Supplement: Multimedia Appendix 5 [file formative_v8i1e55137_app5.docx]

**Multimedia Appendix 5**

# **Title:** Leveraging Implementation Science at the Early-Stage Development of a Novel Telehealth-Delivered Fear of Exercise Program to Understand Intervention Feasibility and Implementation Potential: Feasibility Behavioral Intervention Study

**Authors:**

Andrea T. Duran^1^, MPhil, PhD; Robin M. Cumella^1^, BA; Miguel Mendieta^1^, BA; Adrianna Keener-Denoia^1^, MA; David López Veneros^1,2^, RN, MA, MPhil; Samantha G. Farris^3^, PhD; Nathalie Moise^1^, MS, MD; Ian M. Kronish^1^, MPH, MD

^1^Center for Behavioral Cardiovascular Health, Columbia University Irving Medical Center, New York, NY, United States

^2^Columbia University School of Nursing, New York, NY, United States

^3^Department of Psychology, Rutgers, The State University of New Jersey, Piscataway, NJ, United States

**Corresponding Author:**

Andrea T Duran, MPhil, PhD

Center for Behavioral Cardiovascular Health

Columbia University Irving Medical Center

622 West 168th Street

New York, NY, 10032

United States

Phone: 1 212 342 4491

Email: [atd2127@cumc.columbia.edu](mailto:atd2127@cumc.columbia.edu)

**Table S1.** Reducing Exercise Sensitivity with Exposure Training implementation determinant themes, codes, and representative quotes categorized by the Consolidated Framework of Implementation Research 2.0 domains and constructs.

| Domain and construct | Theme | Code(s) | Representative quotes from exit interviews |
| --- | --- | --- | --- |
| **Innovation** | | | |
| **Innovation design** | Interoceptive exposure dose | Duration and frequency | “I guess [the interoceptive exposure activity] could have been a little longer. Like duration slash frequency…Once you got pumped up it just kind of stopped, you know what I mean?” ID_439  “The basic two sessions what we did for the walking exercise, I think it gives them some confidence, but probably for some people it doesn't give enough…Maybe a little bit longer, more, more walking sessions involved (for patients who) were close to their event...but you know, it's scary.” ID_451 |
|  |  | Challenge and intensity | “The walking part, I think was a little slow for me. It was a little slow. I could have walked a little bit faster… The pace part is the only thing I would change.” ID_449  “The only thing that I would change is having some higher level challenge if possible. Or the option of it.” ID_439 |
|  | Clarity of intervention instructions | Clear communication | “The study team was … good at explaining everything and going over the directions.” ID_439  “[The study team] communication was great. They talked slow. Clearly. If I didn't understand something, they would explain it to me so that I can understand it...they did a great job explaining exactly what entitled, what is expected, what is not expected. It was great. I had no complaints.” ID_449 |
|  |  | Clear presentation | “So, the answers and questions and the booklets was simple and easy to read. It was good.” ID_439 |
|  | Perception of intervention design and equipment | Intervention equipment approval | “I like … that I had the Fitbit and I could record everything.” ID_439  “I think everything in terms of equipment, I think that…it was enough… If they would like, you know, send an iPad over that might be too much.” ID_439 |
|  |  | Intervention design approval | “[The intervention] was pretty straightforward, and it was cost effective.” ID_439  “[The intervention] was nice. It was wonderful and interesting. I learned a lot.” ID_449  “I'm satisfied with the experience. I think it's a good program.” ID_451 |
| **Innovation adaptability** | Tailor intervention to participant needs and preferences | Tailor intervention design to participant | “We do the pace according to each individual person. Some people can walk slow, some people can walk medium, some people can walk fast, some people can walk [inaudible]. I would say, according to the person's pace, it may help.” ID_449  “Make (the pace) individual to each client. What they can do and what they can’t do.” ID_449 |
|  |  | Opportunity for participant choice | “I mean my computer doesn't have a webcam, but I could have got a webcam so I could have seen it on a bigger screen, or put it onto my TV because I think it was only like phone or tablet that were the choices. So that could have had an impact for some people.” ID_439  “Like if you had the option of someone who could do like a 10 minute program versus a six minute or 12 minute, whatever if they wanted to do it...if people are interested.” ID_439 |
|  |  | Scheduling flexibility | “What did I like the least about it? Maybe the timing, you know, the timing is never going to be what I want, you know?... Night would be great, but you can’t really pick your time.” ID_449 |
| **Innovation complexity** | Ease of using intervention materials and equipment | Ease of using intervention materials | “The homework was easy, you know, write down how you feel. That's it. That's easy. And you, you think about it, it's nothing to think about. It's not hard. It's, write everything down. It's like writing a diary or journal.” ID_449 |
|  |  | Ease of using intervention equipment | “Study equipment was ... useful and … everything worked, so that was good.” ID_439 |
|  | Difficulty of virtual delivery modality | Visibility | “I thought it was hard to see some of the prompts and it appeared that it was like kind of unclear to see...on the screen. And then because the phone, I used on my phone and it was like kind of small.” ID_439  “It's difficult to adjust everything and keep the walking course in view. Hard to find the right angles.” ID_439 |
| **‘** |  | Usability | “If you are not a computer person, the Zoom can be challenging especially on the phone.” ID_451  “Setting up for the walking activity would be easier on a bigger screen.” ID_439 |
| **Innovation recipient role (characteristics)** | | | |
| **Capability** | Self-efficacy | Confidence | “I can tell (patients) that it’s a good thing that they participate in because it can give them a boost of confidence.” ID_451 |
|  |  | Belief in ability to achieve task | “And I like the fact that, yes, I can do this. Even if I say to myself, ‘No, I can't’, I can. Even if for 15 minutes, even if for 20 minutes, I learned that I can do this. I can actually walk; I can actually get up and just do this.” ID_449 |
| **Motivation** | External motivation | Accountability | “You know it's good to have somebody sit here and say ‘OK twice a week’ – I know I'm going to be there as opposed to ‘um, I gotta do this on my own’. You start and then you just give it up. But when you have somebody that you know is going to read your work or it's going to need your work to give to somebody else and to pass it along, it makes you want to do it more….” ID_449 |
|  |  | Incentive | “[The Fitbit] was something like level of like additional external motivation… even though the intervention and stuff was kind of short, it was still good to have some other source of tracking of what your physical activity was.” ID_439  “Some people just need that incentive. Other than that, I’m good, I get up every day and I do this, I do this. But sometimes it's just not enough. And you understand that it's not enough.” ID_449 |
| **Opportunity** | Convenience of virtual delivery modality | Convenience and Home-based setting | “What I liked is that it is convenient because you can do it on your own schedule...and you can do it from home.” ID_451  “It makes it a lot easier when you have the space to do your exercise at home. It's more convenient, more comfortable.” ID_449  “… You don't have to go anywhere.” ID_439 |
| **Need** | Appropriate Patient Population | Sedentary | “[Patients] got so used to just sitting here, laying in the bed, laying in the bed, and not moving ... those are the people that really, really do need real help.” ID 449 |
|  |  | Recent Cardiac Event | “I think [the intervention is] the first step forward after a heart attack.” ID_451  “…If you were in the beginning stages or it was a more recent cardiac event, I think [the intervention] would be more appropriate for someone who is somewhat reluctant.” ID_439 |
| **Inner setting** | | | |
| **Available resources (space)** | Home environment capacity | Uneven surface | “Specially an even surface… because unfortunately in my current apartment I have to have a carpet on the floor... so, it was hard to just set it up on a, you know, just on the carpet. In a normal situation, if I don't take into consideration that uneven surface it would not be a problem.” ID_451 |
|  |  | Insufficient space | “The challenge that I had, is more of a, you know, the space issue for setting up the course. The only challenge was that... maybe it was just me…I felt kinda dizzy…you’re turning back and forth. So probably, doing that for longer walking course [would be helpful]. Because, I think, for me it was a little bit short...a couple of steps and a couple of steps.” ID_451 |
| **Available resources (funding, materials and equipment)** | Access to intervention materials | Cost and access | “[The intervention] was cost effective. You don't have to pay any money.” ID_439  “I got everything in a timely manner, all the materials received.” ID_439 |
| **Access to knowledge and information** | Quality of intervention support | Team accessibility | “The study team was very accessible… very supportive.” ID_439 |
|  |  | Quality of interactions | “They gave me time to say what I wanted to say. They gave me time to answer questions. They gave me time to ask questions, and if I didn't know something, they gave me time to volunteer stuff.” ID_439  “They're very patient. Very polite, so yes, I would definitely 100% recommend them.” ID_449 |
